# Mitochondrial function in development and disease

**DOI:** 10.1242/dmm.048912

**Published:** 2021-06-11

**Authors:** Marlies P. Rossmann, Sonia M. Dubois, Suneet Agarwal, Leonard I. Zon

**Affiliations:** 1 Department of Stem Cell and Regenerative Biology, Harvard University, Cambridge, MA 01238, USA; 2 Stem Cell Program and Division of Hematology/Oncology, Boston Children's Hospital, Harvard Medical School, Howard Hughes Medical Institute, Boston, MA 02115, USA; 3 Division of Hematology/Oncology, Boston Children's Hospital, Harvard Medical School, Boston, MA 02115, USA

**Keywords:** Mitochondrial diseases, Mitochondrial fusion and fission, Mitochondrial unfolded protein response, Mitophagy, mtDNA heteroplasmy and inheritance, mtDNA-mediated innate immune response

## Abstract

Mitochondria are organelles with vital functions in almost all eukaryotic cells. Often described as the cellular ‘powerhouses’ due to their essential role in aerobic oxidative phosphorylation, mitochondria perform many other essential functions beyond energy production. As signaling organelles, mitochondria communicate with the nucleus and other organelles to help maintain cellular homeostasis, allow cellular adaptation to diverse stresses, and help steer cell fate decisions during development. Mitochondria have taken center stage in the research of normal and pathological processes, including normal tissue homeostasis and metabolism, neurodegeneration, immunity and infectious diseases. The central role that mitochondria assume within cells is evidenced by the broad impact of mitochondrial diseases, caused by defects in either mitochondrial or nuclear genes encoding for mitochondrial proteins, on different organ systems. In this Review, we will provide the reader with a foundation of the mitochondrial ‘hardware’, the mitochondrion itself, with its specific dynamics, quality control mechanisms and cross-organelle communication, including its roles as a driver of an innate immune response, all with a focus on development, disease and aging. We will further discuss how mitochondrial DNA is inherited, how its mutation affects cell and organismal fitness, and current therapeutic approaches for mitochondrial diseases in both model organisms and humans.

## Abstract (precis)

**Summary:** Mitochondria have a plethora of functions beyond metabolism. This Review discusses the emerging and multifaceted roles of mitochondria in different model organisms and human disease biology.

## Introduction

The word ‘mitochondria’ stems from the Greek words ‘mitos’ for ‘thread’ and ‘chondro’ for ‘grain’. It was coined by the microbiologist Carl Benda in 1898 to reflect the organelles’ morphological diversity, as he observed mitochondria both as threads and granular structures (Benda, 1898). The enormous plasticity of mitochondria was later confirmed by George Emil Palade's pioneering electron microscopy studies (Palade, 1953). A few years earlier, the introduction of differential centrifugation (Bensley and Hoerr, 1934) allowed Albert Claude to isolate intact mitochondria, and demonstrate that succinoxidase and cytochrome oxidase were exclusively mitochondrial (Claude, 1946; Hogeboom et al., 1946), establishing mitochondria as the center of aerobic respiration (Pagliarini and Rutter, 2013). Indeed, >90% of the cell's adenosine triphosphate (ATP) is generated in mitochondria (Harris and Das, 1991) by oxidative phosphorylation (OXPHOS), the complex biochemical process in which electrons are transferred from NADH and FADH_2_ to O_2_ via electron carriers within the electron transport chain (ETC). The latter generates the proton motive force across the inner mitochondrial membrane (IMM) that is harnessed by complex V, the *F*_1_*F*_0_ ATP synthase, to generate ATP from adenosine monophosphate (AMP).

Their ATP production led to mitochondria being described as the ‘powerhouses’ of the cell. As such, mitochondria use different fuels, the classic ones being pyruvate or fatty acids that are channeled through the Krebs [or tricarboxylic acid (TCA)] cycle, but also glutamine and branched-chain amino acids (Spinelli and Haigis, 2018). However, mitochondria play a multitude of roles beyond ATP generation: they are biosynthetic hubs for nucleotides, amino acids, lipids, the urea cycle, gluconeogenesis and ketogenesis, heme and iron-sulfur clusters, and they regulate non-shivering thermogenesis. Mitochondria also re-purpose waste generated by different cellular pathways, such as ammonia and hydrogen sulfide. Furthermore, the discovery that mitochondria, through cytochrome c release, regulate caspase activation and cell death (Liu et al., 1996) started the exploration of their role in the release of reactive oxygen species (ROS) induced by hypoxia to activate an adaptive transcriptional response. In the other direction, the assembly of signaling complexes comprised of A-kinase-anchoring protein and protein kinase A on the outer mitochondrial membrane (OMM) positions mitochondria as targets of converging cellular signaling pathways. Mitochondria also collaborate with other organelles via membranous contacts called mitochondria-associated membranes (MAMs); for example, MAMs shared with the endoplasmic reticulum (ER) are necessary for intracellular calcium homeostasis (Chandel, 2014). Lastly, their ancient origins as an ‘invader’ (see Box 1, ‘Origins of mitochondria’) might relate to the recent observation that mitochondrial DNA (mtDNA) released into the cytosol can elicit an immune response by its symbiotic host.

**Box 1. Origins of mitochondria**

Mitochondria are double-membrane-bound organelles found in nearly all eukaryotic cells, a well-known exception being the erythrocytes of most vertebrates. They likely originated ∼2 billion years ago. A hypothesis of eukaryogenesis that has recently been strengthened by experimental data posits that an archaeal host cell merged with an alphaproteobacterial endosymbiont – the future mitochondrion – to evolve into the first eukaryotic cell that was facultatively aerobic and thus could adapt to and exploit the rising oxygen levels in the environment (Roger et al., 2017; Sagan, 1967). Just recently, such an archaeal host named *Lokiarchaeota*, belonging to the clade *Asgard* – both names from Norse mythology – was first discovered by metagenomic analyses (Spang et al., 2015; Zaremba-Niedzwiedzka et al., 2017). Subsequently, a modern-day version of such an archaeal host could be isolated from a deep-sea sediment core and cultivated in a 12-year experimental feat as *Candidatus Prometheoarchaeum syntrophicum* – named after Prometheus, the god from Greek mythology who created humanity from mud (Imachi et al., 2020).

As a consequence of their origin, mitochondria harbor their own DNA (mtDNA), and replication and transcription follow rules that are different from those governing nuclear DNA (nDNA; see Box 2 and the ‘mtDNA mutations and inheritance’ section). Being much more stable than nDNA, mtDNA is of great importance for forensic medicine and anthropology. The retrieval of complete mtDNA sequences was key to identifying the remains of the last Russian tsar Nicholas II and his family (Ivanov et al., 1996; Rogaev et al., 2009). mtDNA haplotype analysis crucially also aided in the reconstruction of overseas expansion and settlement of the Vikings from Scandinavia (Krzewinska et al., 2015).

**Box 2. Mitochondria basics**

Human mtDNA is a circular double-stranded DNA (dsDNA) molecule of 16,569 bp in length (Anderson et al., 1981) (Fig. 2A). It encodes 13 proteins for essential hydrophobic subunits of electron transport chain (ETC) complexes I, III, IV and ATP synthase, as well as 22 transfer RNAs (tRNAs) and two ribosomal RNAs (rRNAs). Thus, even though mtDNA only encodes a relatively small number of the ∼100 proteins involved in oxidative phosphorylation (OXPHOS), mitochondrial gene expression is essential, with its loss leading to a breakdown of OXPHOS (Larsson et al., 1998). The process of chemiosmosis accomplished by the OXPHOS system yields a mitochondrial membrane potential (ΔΨ_m_) of 150-200 mV across only ∼5 nm of inner mitochondrial membrane, with a resulting field strength (∼30 million V/m) comparable to that of a discharging lightning bolt. Maintaining this ΔΨ_m_ is crucial for cell viability. A core genome encoding genes of the ETC was retained in all mitochondria capable of OXPHOS, which is thought to allow mitochondria to quickly react to changes in ΔΨ_m_ (Lane and Martin, 2010).

To translate the 13 ETC mRNAs, mitochondria contain their own translation machinery, with all the RNA and protein components encoded by mtDNA and nDNA, respectively. The mitochondrial genetic code differs from the nuclear: in human mtDNA, AUA and AUG code for Met, UGA – instead of being a STOP codon – codes for Trp, and AGA and AGG serve as stop codons rather than coding for Arg as in the nuclear genome (Anderson et al., 1981; Barrell et al., 1979). In mouse mtDNA, only UAA serves as a STOP codon (Bibb et al., 1981). In addition to the 13 protein-coding genes, mtDNA harbors a growing list of short open reading frames (sORFs) that encode mitochondrial-derived peptides with systemic functions (Kim et al., 2017). The first sORF identified resides within the rRNA gene *MT-RNR2* and encodes humanin. The complementary DNA (cDNA) for humanin was retrieved in a screen for overexpressed factors that protect neurons from death caused by several early-onset familial AD genes (Hashimoto et al., 2009; Hashimoto et al., 2001a; Hashimoto et al., 2001b). More recently, an *in silico* search identified the sORF *MOTS-c* within the 12S rRNA gene *MT-RNR1* (Lee et al., 2015). Interestingly, *MOTS-c* RNA seems to be exclusively translated in the cytoplasm and constitutes a retrograde signaling pathway in that, upon starvation or oxidant stress, it accumulates in the nucleus where it binds to antioxidant response elements to activate over 1000 genes (Kim et al., 2018). MOTS-c primarily targets skeletal muscle to regulate insulin sensitivity, probably downstream of AMP kinase activation. Indeed, 1 week of MOTS-c treatment restored insulin sensitivity of 12-month-old (aged) to that of 3-month-old (young) mice (Lee et al., 2015).

Each mitochondrion contains 1-15 mtDNA molecules (Bogenhagen and Clayton, 1974; Borst and Kroon, 1969; Nass, 1969; Satoh and Kuroiwa, 1991) and diploid cells contain between 2400 and 6000 mtDNA molecules/cell (Bogenhagen and Clayton, 1974; Shmookler Reis and Goldstein, 1983). This number varies with cell type and tissue (Veltri et al., 1990) and can increase, for example, in muscles performing physical activity (Holloszy, 1967). Despite their abundance, mtDNA molecules only rarely recombine (Howell, 1997), although, interestingly, they can be forced to do so (Ma and O'Farrell, 2015).

The ∼5 μM long circular mtDNA molecule is highly compacted into an aggregate called a nucleoid to fit into the ∼0.5 μM wide mitochondrion (Gustafsson et al., 2016; Nass, 1966). Recent stimulated-emission-depletion super-resolution microscopy data indicate that, in human primary fibroblasts, a single nucleoid contains ∼1.4 mtDNA molecules on average (Kukat et al., 2011). Although devoid of histones, mtDNA is still protein coated, mainly with mitochondrial transcription factor A (mtTFA or TFAM) (Alam et al., 2003; Kaufman et al., 2007) and others (Bogenhagen et al., 2008; Garrido et al., 2003). mtDNA lacks introns and contains just one non-coding control region (NCR; also called D-loop region) harboring regulatory elements including the displacement loop (D-loop), which is essential for replication and transcription (Gustafsson et al., 2016). Transcription by the mitochondrial DNA-directed RNA polymerase (POLRMT), assisted by TFAM and TFB2M (mitochondrial transcription factor B2), produces a near-genome-length polycistronic primary transcript that originates from the light-strand promoter (LSP) within the NCR and is enzymatically processed into the mature mRNA, tRNA or rRNA. Several models of mtDNA replication have been proposed, the favored being the ‘strand displacement model’ (Fig. 2B), in which, after dsDNA unwinding by the DNA helicase Twinkle (TWNK), replication starts from the origin of replication (O_H_) of the heavy strand (H-strand; enriched in guanines) within the D-loop, using shorter transcripts originating from the upstream LSP as an RNA primer. The newly synthesized H-strand displaces the parental H-strand, which gets coated by mitochondrial single-stranded DNA-binding protein (mtSSB). Light-strand (L-strand; enriched in cytosines) replication depends on that of the H-strand and starts at its own origin of replication (O_L_), primed again by POLRMT. mtDNA is continuously replicated, independent of the cell cycle and thus even in non-dividing cells, in a process called ‘relaxed replication’. However, tissue-specific differences in the mode of mtDNA replication exist (Herbers et al., 2019). There might be several other polymerases (Krasich and Copeland, 2017), but mtDNA is mainly replicated by the nuclear-encoded DNA polymerase γ (Polγ;comprising the catalytic POLG and processivity POLG2 subunits) that has 3′-5′ exonuclease (proofreading) activity. mtDNA undergoes less repair compared to nDNA, with the best characterized and probably main mitochondrial pathway being base excision repair, which removes oxidized bases (Kazak et al., 2012). Mutations in components of the basic mtDNA replication machinery cause a large number of mitochondrial diseases, with ∼300 and ∼30 pathogenic mutations identified in *POLG* and *TWNK*, respectively (Copeland; Fratter et al., 2010; Goffart et al., 2009).

In this Review, we will provide the reader with a foundation of the mitochondrial ‘hardware’, the mitochondrion itself, its DNA, mitochondrial dynamics and mitochondria's communication with their ancient host, with a focus on how these mechanisms affect development, disease and aging. We will only cover mitochondrial metabolism as it is relevant to these processes. Excellent recent reviews have extensively summarized mitochondrial metabolism (Lee et al., 2020b; Martinez-Reyes and Chandel, 2020; Spinelli and Haigis, 2018). We will examine the inheritance of mtDNA, mitochondrial fission and fusion, and cellular responses to mitochondrial dysfunction such as mitohormesis, the mitochondrial unfolded protein response (UPR^mt^), ‘piecemeal’ mitophagy or that of the entire organelle, and the immune response the cell directs towards mtDNA escaped into the cytosol.

## Mitochondrial dynamics

**Fig. 1.**
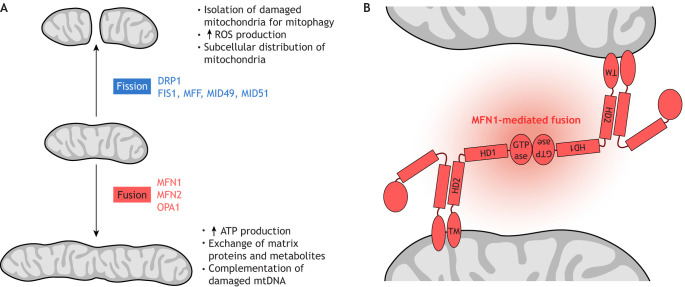
**Mitochondrial fusion and fission.** (A) Factors involved in these processes and effects on mitochondrial activity. (B) Mitofusin 1 (MFN1)-mediated fusion between two outer mitochondrial membranes. Mitofusins are dynamin-related GTPases essential for mitochondrial fusion, which in turn is crucial for physiological mitochondrial function. Importantly, fusion allows complementation of damaged mtDNA (Nakada et al., 2001). Fusion defects cause neurologic disease (see Table 1). MFN1 is comprised of an N-terminal GTPase domain and two coiled-coil heptad-repeat regions (HR1 and HR2) that are separated by two adjacent small transmembrane domains. This model is based on crystal structures of a truncated version of MFN1 lacking the C-terminal part of the HR1 domain, the transmembrane domain (TM) and the N-terminal part of the HR2 domain (see Cao et al., 2017; Qi et al., 2016). ATP, adenosine triphosphate; GTPase, guanosine triphosphate hydrolysis domain; HD1, helical domain 1, HD2, helical domain 2; mtDNA, mitochondrial DNA; ROS, reactive oxygen species.

Mitochondria are mobile and can change in number and morphology, thus forming a dynamic, highly interconnected tubular network, or occurring as isolated or clustered organelles. The subcellular localization of mitochondria often reflects local metabolic demands, and in turn dictates the local ATP:adenosine diphosphate (ADP) ratio, resulting in intracellular energy gradients (Alshaabi et al., 2020). Mitochondria also interact with other membranous cellular structures and organelles. To enable this extraordinary plasticity, mitochondria undergo continuous remodeling by fusion and fission (Fig. 1A).

**Table 1.**
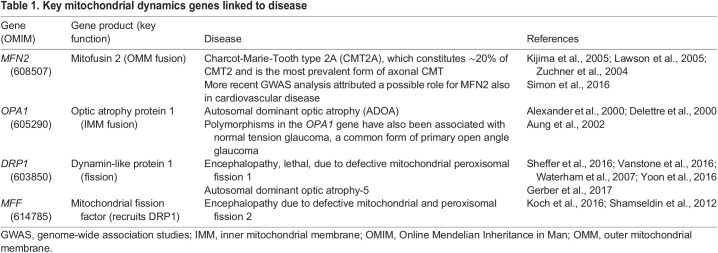
**Key** **mitochondrial dynamics genes linked to disease**

Mitochondria have two membranes: the OMM, which envelops the entire organelle, and the IMM. The IMM is further subcompartmentalized into the inner boundary membrane (IBM), juxtaposing the OMM, and cristae, which are connected to the IBM via narrow cristae junctions and protrude into the mitochondrial matrix, expanding the IMM surface area. In addition to the ETC and the *F*_1_*F*_0_ ATP synthase, cristae contain molecular machinery for protein translocation, metabolite exchange, mitochondrial morphology, protein translation, iron-sulfur biogenesis and protein degradation. The membrane potential (ΔΨ_m_; Box 2, ‘Mitochondria basics’) across the IMM provides the energy required for ATP synthesis. Using new-generation confocal and stimulated-emission-depletion microscopy, the ΔΨ_m_ was recently determined to be different not only between the IBM and cristae but also between different cristae, suggesting that cristae function as independent interconnected ‘batteries’ (Wolf et al., 2019). Restoring proper IMM architecture could therefore be a vital therapeutic approach for diseases with impaired cristae structure such as autosomal dominant optic atrophy (ADOA), ischemia and aging.

### Exploiting tethering mechanisms to treat disease

Mitochondrial fusion and fission are conserved from yeast to humans and are tightly regulated by mitofusin (MFN) 1 and 2, which control OMM fusion, optic atrophy protein 1 (OPA1), which regulates IMM fusion, and by the dynamin-like protein 1 (DLP1; also called DRP1) and its recruiters FIS1, MFF, MID49 and MID51, which regulate fission. Specifically, post-mitotic tissues such as neurons, cardiac and skeletal muscle that are not able to dilute mutated or otherwise faulty mitochondria via cell division rely on compartmentalizing damaged mitochondria for subsequent removal by mitophagy. These tissues are affected by several human diseases that result from impaired mitochondrial dynamics (Table 1).

MFN1 and MFN2 are highly homologous dynamin-related GTPases (Santel et al., 2003) embedded in the OMM. Both proteins are comprised of an N-terminal GTPase domain and two coiled-coil heptad-repeat regions that are separated by two adjacent small transmembrane domains. Despite this high degree of homology, both are individually essential for embryonic development (Chen et al., 2003) and tissue homeostasis (Chen et al., 2007, 2010 and below), indicating different roles for MFN1 and MFN2 in fusion and beyond. Indeed, while MFN1 – together with OPA1 in the IMM – is central to the fusion process, the role of MFN2 in fusion appears to be more complex. Besides its involvement in the actual membrane fusion process, MFN2 also promotes the tethering of mitochondria to other organelles, such as the ER.

Mitofusins are thought to fuse adjacent mitochondria through a combination of oligomerization and GTP hydrolysis (Chen et al., 2003; Koshiba et al., 2004), but the exact mechanism remains elusive. One model posits that mitochondrial tethering – the first step towards fusion – is achieved by the trans-dimerization of mitofusins on different mitochondria via anti-parallel binding of their C-termini (Koshiba et al., 2004). Extending these observations, Franco and colleagues observed that mitofusins exist in two conformations, tethering-constrained/inactive or tethering-extended/active, that are mediated by the balance between intra- or intermolecular interactions, respectively (Franco et al., 2016). In elegant competition experiments, the authors introduced a recombinant MFN2 minipeptide (MP1^Gly^) into cultured neurons from mouse pups expressing a human pathogenic *MFN2*^T105M^ allele with a mutation in the GTPase domain that produces a fusion-defective MFN2 protein. These mice were developed as a model that closely recapitulates the human neurodegenerative disease Charcot-Marie-Tooth (CMT) type 2A (CMT2A) associated with early-onset foot and leg muscular atrophy, scoliosis and ataxia (Detmer et al., 2008). The minipeptide outcompeted dominant intramolecular interactions due to the defective GTPase domain and thus promoted fusion. This reversed the mitochondrial morphological abnormalities of the CMT2A model, including fragmentation and clumping (Franco et al., 2016). This work is not only the first to suggest that the structural flexibility of MFN2 itself is key to mitochondrial dynamics, but also described the first synthetic activator of mitochondrial fusion. A more recent contrasting model (Fig. 1B) shows that, rather than dimerizing via its C-termini, MFN2 homo- or heterodimerizes (with MFN1) via its GTPase domains (Cao et al., 2017; Daumke and Roux, 2017; Li et al., 2019; Qi et al., 2016; Yan et al., 2018a).

Zhou and colleagues found that the mitochondrial fusion defect in mouse embryonic fibroblasts (MEFs) lacking endogenous *Mfn2* but overexpressing the *MFN2*^R94Q^ variant that, like *MFN2*^T105M^, contains a mutation in the GTPase domain, could also be partially rescued by the MP1^Gly^ minipeptide, but only if functional MFN1 was present. This finding led them to hypothesize that a lack of functional MFN2 could be mitigated by raising MFN1 expression levels. Indeed, the CMT2A-associated phenotypes in mice expressing a transgene of the dominant-negative *MFN2*^R94Q^ variant from a pan-neuronal promoter were nearly completely rescued by a second neuronal transgene overexpressing *MFN1* (Zhou et al., 2019). These results indicate that the canonical function of MFN2 in fusion, and not other MFN2-specific functions, causes the clinical symptoms in CMT2A. Furthermore, they invite speculation that raising MFN1 levels could be a therapeutic strategy, possibly beyond the treatment of CMT2A, i.e., for other CMT and neurodegenerative diseases in which mitochondrial dynamics might play a role, including amyotrophic lateral sclerosis (ALS), Huntington's disease (HD), Parkinson's disease (PD) and Alzheimer's disease (AD) (Burté et al., 2015; Wang et al., 2018a). Another strategy to identify fusion activators used a high-throughput chemical screen that uncovered leflunomide, an inhibitor of the mitochondrial pyrimidine *de novo* synthesis enzyme dihydroorotate dehydrogenase, to promote mitochondrial fusion in wild-type MEFs and those carrying single knockouts of either *Mfn1* or *Mfn2* (Miret-Casals et al., 2018).

### Mitochondrial dynamics and organelle crosstalk

Mitochondria interact with the ER (Bernhard and Rouiller, 1956; Rizzuto et al., 1998; Vance, 1990) and other membranous structures in the cell, such as the plasma membrane, the Golgi, lysosomes, peroxisomes (Sugiura et al., 2017), endosomes, melanosomes (Daniele et al., 2014) and lipid droplets in brown adipose tissue (BAT) (Benador et al., 2018; Blanchette-Mackie and Scow, 1983). These interactions predominantly occur in the juxtanuclear region and vary in number between cells from only a few to hundreds of contacts that can last from just seconds to minutes (Valm et al., 2017).

The best studied mitochondria-organelle interaction is that with the ER (for excellent reviews see Giacomello and Pellegrini, 2016; Simmen and Herrera-Cruz, 2018). The first evidence that mitochondria-ER contacts (MERCs) are functionally relevant came from biochemical fractionation experiments that suggested the exchange of lipids between the ER and mitochondria (Vance, 1990). Later, MERCs were also found to mediate Ca^2+^ transfer (Rizzuto et al., 1998). A small fraction of MFN1 and, more so, MFN2 is found in MAMs (de Brito and Scorrano, 2008; Poston et al., 2013), but only MFN2 has been shown to tether mitochondria to the ER (Chen et al., 2012; de Brito and Scorrano, 2008; Naon et al., 2016, 2017; Sebastian et al., 2012; Sugiura et al., 2013). However, there is some debate about whether there are more or fewer MERCs upon MFN2 loss (Cosson et al., 2012; Filadi et al., 2015, 2017; Leal et al., 2016).

MERCs are also involved in mitochondrial fission. Observations in both yeast and mammalian cells showed that most mitochondrial fission events initiate with constriction at MERCs (Friedman et al., 2011). Mechanistically, activation of the formin protein INF2 leads to actin polymerization, which is hypothesized to generate the necessary force for the initial mitochondrial constriction to which the DRP1 GTPase is recruited. DRP1 then, via its GTP hydrolysis activity, induces further constriction to drive fission forward (Korobova et al., 2013; Smirnova et al., 2001). It is interesting to note that *INF2* mutations cause the degenerative kidney disease focal and segmental glomerulosclerosis (Brown et al., 2010) as well as CMT disease (Boyer et al., 2011).

The final mitochondrial fission steps that lead to the actual scission are still unclear. Recently, Nagashima et al. have shown an essential role for trans-Golgi network (TGN)-derived vesicles. Downstream of DRP1, these TGN vesicles are recruited to MERCs via phosphatidylinositol 4-phosphate [PI(4)P]-enriched microdomains. This depends on the activity of the Arf1 GTPase and the Arf1 effector PI(4)-kinase-III-b [PI(4)KIIIb] that generates PI(4)P (Nagashima et al., 2020). The authors speculated that PI(4)P recruits adaptor proteins that in turn drive the assembly of an actin-polymerizing machinery relevant for scission. In addition, a role for dynamin 2 in the final mitochondrial division steps downstream of DRP1 has been suggested (Lee et al., 2016), which, however, remains disputed (Fonseca et al., 2019; Kamerkar et al., 2018).

MERCs are also thought to function as key signaling hubs in inflammasome activation (Zhou et al., 2011), autophagosome formation (Hamasaki et al., 2013) and ROS signaling (Booth et al., 2016). Upon ER stress, hypoxia or starvation, mitochondria and ER move closer together, whereas excess glucose lets them move further apart (Prasad et al., 2016; Rieusset, 2018). Both reduced and increased MERC formation have been implicated in tumor growth (Cárdenas et al., 2016; Raturi et al., 2016), and have been observed in neurodegenerative diseases (Simmen and Herrera-Cruz, 2018).

### Fusion-unrelated functions of MFN2

#### MFN2, MAMs and axonal transport

A novel and exciting role of MFN2 at neuromuscular synaptic junctions (NMJs), which decline in aging-related sarcopenia as well as in ALS-related skeletal muscle wasting, has been elucidated by Wang and colleagues (Wang et al., 2018a). The authors found that MFN2's function in tethering ER and mitochondria is essential for carrying calpastatin to NMJs, where it executes its key protective effects by inhibiting calpain. The *Mfn2*^ActA^ mutant, which can fuse mitochondria but cannot form ER-mitochondria tethers (de Brito and Scorrano, 2008), was unable to increase calpastatin axonal transport. In contrast, wild-type *Mfn2* or the *Mfn2*^IYFFT^ mutant that can form ER-mitochondria tethers but cannot fuse mitochondria increased calpastatin axonal transport. Along the same lines, forced expression of *Mfn2* in the spinal cord in the ALS-modeling *Sod1*^G93A^ transgenic mice rescued NMJ loss and muscular atrophy and delayed overt disease onset by 60 days, while mitochondria were still fragmented. In addition, overexpression of *Mfn2* in the spinal cord sustained the skeletal muscle weight of 22-month-old aged mice at the levels of those of young mice, confirming that mildly upregulated neuronal MFN2 levels protect NMJs from aging (Wang et al., 2018a). Interestingly, MFN2 dysfunction in the *MFN2*^R94Q^ overexpression CMT2A model also led to altered axonal transport of mitochondria, and pharmacologically reinforcing MAM function or ameliorating ER stress could partially restore wild-type mitochondrial properties and improve physical performance of these mice (Bernard-Marissal et al., 2019). *MFN2* mutations and deficiency are not only found in CMT2A, but also associated with ALS, AD and aging. It is thus likely that MFN2 has a protective effect on synapses in a wide range of muscular and neurodegenerative diseases. As a case in point, upregulation of calpastatin seems to be protective in mouse models of PD (Diepenbroek et al., 2014; Yang et al., 2013), and therefore MFN2 might function through a similar mechanism here. Furthermore, because *Mfn2* ablation triggers ER stress in many systems (Schneeberger et al., 2013; Sebastian et al., 2012), it would be interesting to see whether ER stress inhibitors also influence the transport of calpastatin. More recent work showed that local accumulation of mitochondria in perivascular astrocyte processes after acute brain injury is required to promote vascularization of the injured region, and also depends on MFN2 and the formation of MERCs. Astrocyte-specific *Mfn2* knockout mice exhibited disrupted MERCs, and their astrocytic mitochondria – despite being functional – did not cluster around the vasculature, thus impairing vascular remodeling following injury. This could be rescued by intracortical delivery of a synthetic linker to enforce MERCs in astrocytes (Gbel et al., 2020).

### Mitochondrial dynamics in development and tissue homeostasis

A causal role for mitochondrial fusion was found in the priming of naïve mouse embryonic stem cells (ESCs). Naïve ESCs can be isolated from the inner cell mass of the pre-implantation embryo/blastocyst at embryonic day (E) 3.5-4.5. Slightly later, at E6-E7.5, primed epiblast stem cells (EpiSCs) reside within the late epiblast layer of post-implantation embryos and have a restricted developmental potential (Weinberger et al., 2016). Interestingly, the interconversion from naïve ESCs to primed EpiSCs is characterized by a marked switch to a highly glycolytic state of energy production and low mitochondrial respiration activity, even though EpiSCs have more mature and elongated mitochondria (Zhou et al., 2012), indicating a mitochondrial contribution beyond respiration. A recent study showed that inducing mitochondrial fusion is sufficient to drive exit from the naïve ESC state (Bahat et al., 2018): in naïve ESCs, acute depletion of mitochondrial carrier homolog 2 (MTCH2), a regulator of mitochondrial apoptosis and essential to promote the transition of hematopoietic stem cells (HSCs) from the quiescent into the cycling state (Grinberg et al., 2005; Maryanovich et al., 2015), resulted in fragmented and respiration-defective mitochondria. Intriguingly, the concomitant failure of *Mtch2^-/−^* naïve ESCs to transition to primed EpiSCs could be rescued by overexpression of *Mfn2* or dominant-negative *Drp1* (Bahat et al., 2018).

In the adult, impaired mitochondrial dynamics cause systemic metabolic consequences, particularly in skeletal muscle, the nervous system and adipose tissue. In humans, both heterozygous and homozygous mutations in the essential IMM fusion gene *OPA1* are clinically associated with myopathy (Amati-Bonneau et al., 2008; Davies et al., 2007; Schaaf et al., 2011; Spiegel et al., 2016). While investigating the function of OPA1 in skeletal muscle, several groups discovered a role in whole-body metabolism. Inducible *Opa1* knockout in skeletal muscle of young mice (4 weeks old) resulted in progressive mitochondrial dysfunction, attenuated age-induced weight gain and muscle atrophy, even though these mice had a higher physical exhaustion limit (Pereira et al., 2017). These mice also had improved glucose tolerance and did not become insulin resistant, neither during aging nor when fed a high-fat diet. Intriguingly, loss of *Opa1* function normalized a pre-existing metabolic imbalance due to a high-fat diet. The metabolic improvements were mediated by increased expression of the cytokine FGF21 specifically in muscle tissue, which was likely induced by ER stress. This resulted in greatly increased plasma FGF21 levels. Importantly, in muscle-specific *Opa1*;*Fgf21* double-knockout mice fed a high-fat diet, this systemic metabolic adaptation was completely reversed and the mice became insulin resistant (Pereira et al., 2017). Tezze and colleagues noticed that expression levels of OPA1, MFN1/2 and DRP1 in muscle of sedentary seniors were lower compared to those in senior sportsmen (Tezze et al., 2017). Moreover, in 5-month-old mice, muscle-specific loss of *Opa1* function was associated with an aging phenotype accompanied by muscle wasting, white hair and kyphosis, hepatic steatosis and inflammation, but improved glucose tolerance. Again, *Fgf21* was starkly upregulated, and additional muscle-specific conditional *Fgf21* knockout completely reverted the aging phenotype. Oxidative stress emanating from deficient mitochondria is a speculated source of ER stress upstream of FGF21, as treatment with a vitamin E analog as an antioxidant prevented *Fgf21* induction and restored the muscle mass to almost normal levels (Tezze et al., 2017). Interestingly, anti-inflammatory treatment with the NF-κB inhibitor sodium salicylate could normalize both FGF21 levels and muscle atrophy in a similar adult muscle-specific *Opa1* loss-of-function model (Rodriguez-Nuevo et al., 2018). Further work will be needed to disentangle the upstream events leading to *Fgf21* induction and the consequences of *Opa1* loss during different adult stages.

In the adult mouse forebrain, ablating the fission-promoting factor *Drp1* also induces *Fgf21* expression, specifically in hippocampal and cortical neurons (Restelli et al., 2018). The authors found eukaryotic translation initiation factor 2α (eIF2α), a central player of the integrated stress response (ISR) (Fig. 3), to be phosphorylated, likely as a consequence of ER stress, amino acid deprivation and heme deficiency in *Drp1*-deficient neurons. When phosphorylated, eIF2α leads to the upregulation of ATF4, a transcription factor known to induce *Fgf21* expression. Based on this and other findings that show *Fgf21* induction in mouse models of frontotemporal dementia (FTD) and prion disease, *Fgf21* may be a suitable marker for neurodegenerative diseases that are accompanied by mitochondrial dysfunction (Restelli et al., 2018).

#### A role for Mfn2 in the hypothalamus-adipose axis

The hypothalamus has a central role in the regulation of whole-body energy homeostasis. Deleting *Mfn2* from murine satiety-promoting pro-opiomelanocortin (POMC)-producing neurons of mice led to extreme obesity, which was associated with mitochondrial morphological changes, including fewer MFN2-dependent MERCs. Specifically, the observed ER stress upon MFN2 reduction rendered POMC neurons resistant to the satiety-promoting hormone leptin. Treating mice with chemical chaperones that relieve ER stress and improve ER function reversed several of these phenotypes (Schneeberger et al., 2013). Of note, POMC neurons constitute only a small subset of all the cells affected by *Pomc*-Cre-mediated recombination (Padilla et al., 2012), such that the effect of *Mfn2* could also be due to a role in other hypothalamic cell populations. In a different study, Dietrich and colleagues observed a role for both *Mfn1* and *Mfn2* in diet-induced obesity by studying the hunger-promoting Agouti-related protein (Agrp) neurons (Dietrich et al., 2013). Using electron microscopy, the authors observed that a high-fat diet promoted mitochondrial fusion-like changes in Agrp neurons. Consequently, *Mfn1* or *Mfn2* loss specifically in Agrp neurons of mice fed a high-fat diet resulted in a decreased firing rate and in leaner mice due to an impaired gain of fat mass. They then used patch-clamp analysis to show that replenishing the dysfunctional Agrp-*Mfn1^−/−^* or Agrp-*Mfn2^−/−^* neurons with ATP rescued their decreased ΔΨ_m_, demonstrating that ATP deficiency caused the electrical activity defect. This suggests that MFN1 and MFN2 are critical for maintaining ATP levels and thus firing activity in Agrp neurons to adapt the organism to systemic metabolic changes and achieving proper fat storage.

*MFN2* expression is downregulated in both adipose tissue of human obese subjects and mice fed a high-fat diet. Of note, *MFN2* expression is also decreased in skeletal muscle of obese and type 2 diabetic patients (Bach et al., 2003), coinciding with more fragmented and smaller mitochondria (Kelley et al., 2002; Bach et al., 2003). Furthermore, just as in the POMC-neuron-specific *Mfn2* knockout mouse model, mice with an adipocyte (adiponectin)-specifc *Mfn2* deletion induced in adulthood gained body weight accompanied by increased plasma leptin and glucose levels, as well as by insulin resistance (Mancini et al., 2019). Thus, disturbed mitochondrial dynamics in adipocytes also affects systemic energy metabolism.

*Mfn2* is expressed at particularly high levels in BAT (Bach et al., 2003). While white adipose tissue (WAT) serves as the primary storage for excess calories as lipids, BAT is critical for thermogenesis to maintain the core body temperature. Both WAT and BAT store lipids in the form of lipid droplets (LDs) (Peirce et al., 2014). In BAT, 60% of mitochondria contact LD membranes (Boutant et al., 2017; Jagerstrom et al., 2009; Pidoux et al., 2011; Rambold et al., 2015). The physical connection between LD and mitochondria is thought to facilitate the transfer of fatty acids for mitochondrial fatty acid oxidation (FAO) (Pidoux et al., 2011; Rambold et al., 2015). Indeed, the number of mitochondria-LD contacts in BAT increases at cold temperatures (Yu et al., 2015). Using adipocyte-specific *Mfn2* knockout mice, Boutant and colleagues showed a critical role for MFN2 in BAT metabolism, tethering LDs to mitochondria via its interaction with the LD scaffolding protein perilipin 1 to maintain proper mitochondrial FAO and OXPHOS (Boutant et al., 2017). Surprisingly, loss of *Mfn2* in BAT protected high-fat diet-fed mice from hepatic steatosis and insulin resistance. This was accompanied by increased ER stress and an upregulation of FGF21 in BAT and in plasma. Thus, knockout of genes involved in mitochondrial fusion in either muscle or adipose tissue seems to converge, via ER stress, on FGF21 expression. However, the resulting systemic metabolic consequences seem to be partially opposite. Regardless, this body of work corroborates that tissue-specific disruption of mitochondrial fusion in skeletal muscle, the nervous system and adipose tissue can affect the regulation of whole-body energy metabolism.

## Mitochondrial homeostasis and quality control

Of the currently catalogued 1136 proteins that reside in mitochondria (Pagliarini et al., 2008; Rath et al., 2021), only 13 are synthesized from mtDNA. The remaining 1123 are encoded by nuclear genes, and, after translation by ribosomes in the cytoplasm, including locally on the mitochondrial surface (Zhang et al., 2016), the polypeptide chains are imported into the mitochondrial matrix via one of five import pathways (Wiedemann and Pfanner, 2017). In mitochondria, these polypeptides are then further proteolytically processed and folded into their active forms. The high protein concentration in the mitochondrial matrix, together with the need for coordinated expression of the mitochondrial and nuclear genomes and the proximity of mtDNA to ETC-derived ROS, can perturb mitochondrial proteostasis (Naresh and Haynes, 2019). Mitochondrial proteostasis is essential to maintain normal cellular function, as several diseases are associated with the misfolding or formation of toxic protein aggregates. The cell maintains proper proteostasis in its mitochondria by one of at least four mitochondrial quality control pathways, namely the mitochondrial unfolded protein response (UPR^mt^), ubiquitin-mediated proteasomal degradation, mitochondria-derived vesicle (MDV)-mediated degradation and mitophagy (Sugiura et al., 2014).

### Mitochondrial quality control by the UPR^mt^

The UPR^mt^ is a retrograde signaling pathway in which the mitochondrion signals local stress back to the nucleus to mount a nuclear transcriptional response aimed at repairing mitochondrial damage and protecting the cell (Fig. 3). The UPR^mt^ is thus a critical component of mitohormesis, the process by which mild mitochondrial stress activates an adaptive response in cells and organisms that confers long-lasting stress resistance (Yun and Finkel, 2014). Similarly to the UPR in the ER (UPR^ER^), the UPR^mt^ increases synthesis of mitochondrial chaperones and reduces mitochondrial protein translation to attenuate the stress caused by misfolded proteins in the mitochondrial matrix. The UPR^mt^ was discovered in mammalian cells that express a mutant insoluble form of the mitochondrial matrix protein ornithine transcarbamylase (OTC) (Martinus et al., 1996; Zhao et al., 2002). Subsequently, many of the UPR^mt^ components were identified in *Caenorhabditis* *elegans* due to its amenability to genetic screens (Benedetti et al., 2006; Haynes et al., 2007; Yoneda et al., 2004). As a key player in nematodes, the basic leucine zipper transcription factor ATFS-1 monitors global import efficiency into mitochondria. ATFS-1 contains both a nuclear localization sequence (NLS) and a mitochondrial-targeting sequence (MTS), with a low net charge that can only be imported into high-ΔΨ_m_ mitochondria (Nargund et al., 2012; Rolland et al., 2019). The fact that ATFS-1 contains both localization sequences positions it optimally for mitochondrial-to-nuclear communication. ATFS-1 is efficiently imported into healthy mitochondria, where it is readily degraded by the protease LONP-1 in the mitochondrial matrix. However, if this import process fails due to mitochondrial stress and an ensuing reduction in ΔΨ_m_, ATFS-1 uses its NLS to translocate into the nucleus, where it activates a broad transcriptional response of ∼500 genes that, among many other processes, promote the production of mitochondrial chaperones and proteases aimed at repairing mitochondrial defects (Lin et al., 2016; Munch and Harper, 2016; Nargund et al., 2015, 2012). Among the most potent inducers of the UPR^mt^ are changes to mitochondrial proteostasis caused, for example, by dysfunctional mitochondrial chaperones or proteases involved in quality control (Yoneda et al., 2004). However, genome-wide RNA interference (RNAi) screens have uncovered perturbations of many mitochondrial processes as inducers of the UPR^mt^ (Rolland et al., 2019).

In contrast to *C. elegans*, the UPR^mt^ pathway is still poorly characterized in mammalian systems. An ATFS-1 homolog has not been identified in mammalian cells. Rather, C/EBP homologous protein (CHOP; DDIT3) and two basic leucine zipper transcription factors, ATF4 and ATF5, are critical in the mammalian UPR^mt^ (Fiorese et al., 2016; Martinus et al., 1996; Quiros et al., 2017; Zhao et al., 2002). All three transcription factors also have established roles within the UPR^ER^ (Yang et al., 2017). In fact, the so-called integrated stress response (ISR), which is activated by a range of (patho-)physiological alterations including ER stress (Pakos-Zebrucka et al., 2016), is required for UPR^mt^ induction in mammals (Munch and Harper, 2016), but dispensable in worms (Baker et al., 2012). The ISR seems to cause the mitochondrial myopathy observed in ‘Deletor mice’ that express a dominant mutation in the mtDNA helicase Twinkle, which is also found in patients with progressive external ophthalmoplegia (PEO) (Table 3). Mechanistically, the mtDNA replication defect in Deletor mice activates mechanistic (mammalian) target of rapamycin (mTOR) complex I (mTORC1), which directly regulates the ISR and also leads to a significant induction of *Atf4*, *Atf5* and other mammalian homologs of UPR^mt^ effectors previously identified in worms, including *Hsp60* (*Hspd1*), *Hsp70* (*Hspa1a/b*), *Lonp1* and *Clpp*. Intriguingly, the mTORC1 inhibitor rapamycin reverses UPR^mt^ activation and the phenotypic hallmarks of the mitochondrial myopathy in these mice (Khan et al., 2017), suggesting that PEO phenotypes are directly caused by aberrant UPR^mt^. Thus, although many studies show specific activation of either the UPR^mt^ or the UPR^ER^, several mammalian proteins and at least one *C. elegans* protein (Runkel et al., 2013) function in both responses, indicating that they are linked. To better understand UPR^mt^ in mammalian species, rather than searching for mammalian homologs to nematode UPR^mt^ players, orthogonal genome-wide approaches might be a fruitful strategy. For example, phenome-wide association studies in mice found that a missense variant in the fumarate hydratase (*Fh1*) gene specifically activates the UPR^mt^ (Wang et al., 2016b). FH1 catalyzes the hydration of fumarate to malate in the TCA cycle. Interestingly, conditional knockout of *Fh1* in the hematopoietic system results in HSCs that are unable to self-renew and differentiate into B and T cells (Guitart et al., 2017), potentially linking TCA cycle integrity and the UPR^mt^ to stem cell fitness.

### The UPR^mt^ in neurodegenerative diseases

Increased levels of UPR^mt^ transcripts have been found in patients with mitochondrial myopathy, cardiomyopathy, aging, glioblastoma, depression, and sporadic or familial AD. Mitochondrial dysfunction is a hallmark of AD (Weidling and Swerdlow, 2020), although whether it is a cause or consequence of the disease is not yet clear. Extracellular senile plaques, one of the main characteristics of AD, are deposits of amyloid beta (Aβ) that arise from abnormal sequential cleavage of amyloid precursor protein (APP). The cortices of patients with mild cognitive impairment, a suggested prodromal stage of AD, as well as of those with mild and moderate AD, and cortices from a transgenic mouse model of AD, had upregulated UPR^mt^ and mitophagy-specific transcripts. This response was conserved in ‘GMC101’ worms expressing a human aggregation-prone Aβ1-42 isoform in muscle cells, which results in age-dependent paralysis (Beck et al., 2016; Sorrentino et al., 2017). Interestingly, inhibition of the UPR^mt^ by RNAi-mediated silencing of *atfs-1* – which also reduces mitochondrial respiration – aggravated Aβ aggregation and paralysis. Conversely, inducing the UPR^mt^ further, for example, by overexpressing *atfs-1*, ameliorated paralysis and prolonged lifespan via reduced Aβ aggregation (Sorrentino et al., 2017). Similar results were achieved with other UPR^mt^-inducing manipulations, such as silencing of the mitochondrial ribosomal protein-encoding gene *mrps-5* (Houtkooper et al., 2013) and treating GMC101 worms with doxycycline (Dox), which inhibits mitochondrial translation (Moullan et al., 2015). This effect seems to be conserved in human cells, as Dox also reduced intracellular Aβ1-42 deposits caused by the Swedish APP mutation in a human neuroblastoma cell line, in a manner dependent on both the ISR and the UPR^mt^ via its mediator ATF4. In GMC101 worms, besides *atfs-1* overexpression, the addition of nicotinamide riboside (NR), a compound that increases NAD^+^ levels, directly improved mobility and lifespan via the UPR^mt^ and mitophagy. Furthermore, a 10-week course of NR reduced Aβ plaques in the brains of an AD mouse model and improved their context-dependent memory (Sorrentino et al., 2017). These results suggest that Aβ aggregation induces the UPR^mt^, possibly due to the alteration of a process such as mitochondrial import, and that boosting proteostasis through further UPR^mt^ activation could be a therapeutic avenue for AD patients.

Interestingly, the opposite outcome of UPR^mt^ activation has been shown for a *C. elegans* model of PD, another neurodegenerative disease for which mitochondrial defects are proposed to play a key role. Here, prolonged overactivation of ATFS-1 in dopaminergic neurons leads to necrosis (Martinez et al., 2017). Interestingly, mutations in *C. elegans pink-1*, the homolog of the mitochondrial kinase PTEN-induced putative kinase 1 (*PINK1*), which together with Parkin is critical for mitophagy (discussed below), activated the UPR^mt^, and this protected the worms from dopaminergic neuron loss (Cooper et al., 2017). A possible explanation could be that although short-term activation of the UPR^mt^ as a mitohormetic response is beneficial, chronic activation can become toxic. A similar observation for a dose-dependent role of the UPR^mt^ has also been made in the context of lifespan (Bennett et al., 2014), indicating that the UPR^mt^ must be tightly regulated. Certainly, a better understanding of these dependencies needs to be gained before therapeutic strategies targeting the UPR^mt^ can be developed.

The UPR^mt^ has also been implicated in the pathogenesis of ALS and HD. Mitochondrial dysfunction has long been thought to play a critical role in HD (Costa and Scorrano, 2012), and several lines of evidence point to impaired protein import/export. Ultrastructural and biochemical observations in cell and animal models of HD illustrate that mutant huntingtin (*HTT*), which contains abnormally expanded CAG repeats that are translated into polyglutamine in the N-terminus of the protein, physically associates with mitochondria (Orr et al., 2008; Yu et al., 2003). Mutant HTT binds to the TIM23 mitochondrial import complex and directly inhibits protein import, leading to neuronal death early during HD pathogenesis in the R6/2 mouse model of HD. Indeed, overexpression of three major TIM23 complex subunits *Timm23*, *Timm50* and *Timm17a* not only rescued the import defect, but also mitochondrial dysfunction and death of primary cortical neurons induced by mutant HTT (Yano et al., 2014). Intriguingly, HD cell cultures, as well as the R6/2 mouse model, exhibited lower levels of the IMM ABC transporter ABCB10 (Fu et al., 2019), which, just as its putative *C. elegans* ortholog HAF-1, is involved in the UPR^mt^ (Haynes et al., 2010; Yano, 2017). Lower ABCB10 levels in fact downregulate the transcription factor CHOP and some of its transcriptional UPR^mt^ targets, including the molecular chaperone *Hsp60* and the protease *Clpp* (Fu et al., 2019). Thus, even though protein import is disrupted in HD neurons, which should trigger the UPR^mt^, the UPR^mt^ is paradoxically inhibited, which is probably at the root of neuronal death in this disease.

A form of UPR distinct from the classic UPR^mt^ and termed IMS-UPR^mt^ is triggered by the accumulation of proteins in the intermembrane space (IMS). Evidence for this pathway first came from studies in the breast cancer cell line MCF-7, in which IMS stress induces estrogen receptor alpha (ERα) phosphorylation and activation in an ROS- and AKT-dependent manner, which in turn upregulates the IMS protease OMI (HTRA2) and NRF1. The latter is required for the expression of several genes of the ETC (Papa and Germain, 2011). Of note, the widely studied *SOD1*^G93A^ mutation that causes 20% of familial ALS cases produces a protein that misfolds and accumulates in both the cytoplasm and the IMS. A transgenic mouse model expressing *SOD1*^G93A^ just in the IMS but not in the cytoplasm takes 1 year longer to develop ALS (Igoudjil et al., 2011). Hence, a similar ERα-dependent activation of a protective IMS-UPR^mt^ could also play a role in ALS, as the incidence of sporadic ALS is much lower in females than in males (Riar et al., 2017).

### When mitochondrial damage cannot be repaired: mitophagy

Protein import efficiency monitors imbalances in mitochondrial homeostasis. Whereas the UPR^mt^ responds to less severe homeostatic defects, leading to organelle repair, severe damage results in mitophagy, the engulfment of the entire mitochondrion by autophagosomes, followed by their transport to and elimination by lysosomes.

Mitophagy occurs in a ubiquitin-dependent or ubiquitin-independent manner, mediated by the autophagy receptor proteins NIP3-like protein X (NIX; BNIP3L), BNIP3 and FUNDC1 (Fig. 4; for a review see Palikaras et al., 2018). The best-studied mitophagy pathway is ubiquitin dependent and involves PINK1 and the ubiquitin ligase Parkin. The genes encoding these proteins, *PINK1* and *PRKN*, are implicated in hereditary juvenile (<20 years of age) or early-onset (20-40 years of age) PD (Kitada et al., 1998; Matsumine et al., 1997; Valente et al., 2004, 2001). *PINK1* and Parkin are genetically linked and constitute the ubiquitin-dependent mitophagy axis (Clark et al., 2006; Narendra et al., 2008; Park et al., 2006). Under homeostatic conditions, PINK1 is imported into the IMM via the TOM/TIM complex, where it is proteolytically cleaved (Harper et al., 2018). A truncated form is then released back into the cytosol and targeted for proteasome-mediated degradation. Thus, because of the continuous import and degradation of PINK1, its levels are normally barely detectable. However, ΔΨ_m_ dissipation, the accumulation of unfolded proteins in the mitochondrial matrix (Jin and Youle, 2013; Pimenta de Castro et al., 2012) or other stresses such as ROS (Xiao et al., 2017) disrupt the mitochondrial import of PINK1 and stabilize it on the OMM. PINK1 phosphorylates Parkin as well as ubiquitin, both of which activate Parkin's E3 ligase activity. Parkin then ubiquitinates several OMM proteins, and the poly-ubiquitin chains in turn serve as additional phosphorylation targets for PINK1, creating a feed-forward loop. The phosphorylated poly-ubiquitin chains trigger the recruitment of ubiquitin-binding adaptor proteins, including optineurin (OPTN), nuclear dot protein 52 (NDP52; CALCOCO2) and possibly p62 (SQSTM1) (Palikaras et al., 2018), which connect ubiquitin-tagged mitochondria to autophagosomes (Fig. 4) (Palikaras et al., 2018). Many mutations in *PINK1* and *PRKN* have been identified in PD patients (for an overview see Pickrell and Youle, 2015) and prevent PINK1-mediated recruitment of Parkin to mitochondria, highlighting that it is the role of PINK1 and Parkin in mitophagy that leads to PD. Of note, only the conditional knockout of *Prkn* in adult mice diminishes dopaminergic neurons in the substantia nigra (Shin et al., 2011), whereas germline *Pink1*/*Prkn* knockout mice have no overt phenotypes (Goldberg et al., 2003; Itier et al., 2003). This suggests that the PINK1/Parkin pathway can likely be compensated for in mice by other mitophagy pathways during development. Indeed, besides Parkin, other E3 ligases acting in parallel mitophagy pathways have recently been identified, such as SMURF1 (Orvedahl et al., 2011), Gp78 (AMFR) (Fu et al., 2013), MUL1 (Yun et al., 2014), SIAH1 (Szargel et al., 2016) and ARIH1 (Villa et al., 2017). In humans, however, the uncompensated high penetrance of juvenile-onset PD caused by *PINK1* or *PRKN* mutations remains as of yet unexplained.

Defects in neuronal mitophagy have also been observed in ALS. Mutations in the ubiquitin binding domain of OPTN, which were identified by targeted sequencing in Japanese patients with ALS (Maruyama et al., 2010), fail to rescue recruitment of the autophagosomal light chain 3 (LC3; MAP1LC3A/B/C) protein and mitophagy in *OPTN*-depleted cultured cells (Wong and Holzbaur, 2014). OPTN's affinity for ubiquitin chains is enhanced by its phosphorylation, and TANK binding kinase 1 (TBK1), recently also found to be mutated in a subset of ALS patients (Cirulli et al., 2015), is a relevant kinase (Heo et al., 2015; Moore and Holzbaur, 2016; Richter et al., 2016). Of note, OPTN also inhibits the innate immune system by negatively regulating TBK1 and thus dampening the activation of interferon regulatory factor 3 (IRF3) in a cell-cycle-dependent manner (Genin et al., 2015). Given the importance of the innate immune system for the development of neurodegenerative diseases (Scheiblich et al., 2020), it will be interesting to determine whether there is crosstalk between these OPTN and TBK1 functions and their roles in mitophagy, contributing to the ALS disease phenotype.

### Crosstalk of mitophagy with mitochondrial fusion and fission

Mitochondrial homeostasis demands a balance between biogenesis, fusion, fission and mitophagy. Thus, it is not surprising that there is crosstalk between these processes. For example, the mitofusins MFN1 and MFN2 are targeted for degradation by the PINK1/Parkin pathway, and this might help prevent damaged mitochondria from fusing with intact mitochondria to selectively eliminate the damaged ones by mitophagy (Tanaka et al., 2010). Indeed, conditional cardiomyocyte-specific *Mfn2* knockout in mice resulted in cardiomyopathy, failure to recruit Parkin to depolarized mitochondria and defective mitophagy (Chen and Dorn, 2013). However, the finding that Parkin is still recruited to mitochondria in embryonic fibroblasts derived from *Mfn1*/*Mfn2* double-knockout mice (Narendra et al., 2008), implies the existence of tissue-specific mitochondrial receptors for mitophagy. Of note, whether the observed cardiotoxicity in the absence of MFN2 is due to impaired mitophagy is not clear, as cardiomyopathy is not observed in Parkin knockout mice (Kubli et al., 2013).

### Physiological roles of mitophagy

The physiological stimuli that induce mitophagy are not known. Many studies have investigated mitophagy by simulating mitochondrial stress with chemicals – mitochondrial poisons or high concentrations of uncoupling reagents (protonophores) that dissipate the ΔΨ_m_ – and by overexpressing *PINK1* and *PRKN* in cancer cell lines. The recently developed ‘*mito*-QC’ and ‘mt-Keima’ reporter mice allow imaging of mitophagy *in vivo*. These mice have revealed tissue-specific variation in mitophagy levels, with high rates in some neuronal cell types, heart, liver, kidney and skeletal muscle, and low rates in spleen and thymus (McWilliams et al., 2016; Sun et al., 2015). Intriguingly, while mitotoxin-induced mitophagy was reduced in *Pink1* knockout mice, basal mitophagy rates were indistinguishable from those in wild-type mice (McWilliams et al., 2018), a result also observed in *Pink1* or *parkin* null flies (Lee et al., 2018). A possible explanation for this finding could be that PINK1/Parkin-induced mitophagy only adds to basal mitophagy, which might require a different mechanism. Alternatively, the findings could be due to different dependencies of mitophagy *in vivo* compared to the *in vitro* approaches that mostly used immortalized cell lines overexpressing these proteins. Future work is needed to elucidate the physiological roles of the PINK1/Parkin axis in mitophagy.

Relatively few studies have investigated the role of mitophagy during development, and the emerging concept is that different cells/tissues have developed different types of ‘programmed mitophagy’. A striking example of physiological mitophagy is the elimination of paternal mitochondria in the zygote. The prevailing thought was that paternal mtDNA is simply diluted because of the relatively small sperm compared to the large egg. However, work in *C. elegans*, *Drosophila* and mammalian species showed that entry of the spermatozoon into the ooplasm triggers mitophagy to actively remove paternal mitochondria (Al Rawi et al., 2011; Politi et al., 2014; Rojansky et al., 2016; Sato and Sato, 2011; Song et al., 2016). This might be important because mtDNA heteroplasmy is deleterious for the developing embryo (see ‘Heteroplasmy and the mitochondrial bottleneck’ section). Alternatively, mitophagy might be a defense mechanism in the fertilized oocyte comparable to that of somatic cells defending themselves against invading bacteria (Levine and Elazar, 2011). Of note, using transgenic mouse strains expressing fluorescently labeled autophagosomes and mitochondria, Luo and colleagues could not observe any mitophagy post-fertilization. Rather, most motile sperm eliminated their mtDNA before fertilization and the rest were diluted by uneven mitochondrial distribution during cell division in the early embryo (Luo et al., 2013). Interestingly, whereas ubiquitination of paternal mitochondria is not required for mitophagy in *C. elegans* (Al Rawi et al., 2011; Sato and Sato, 2011), cow and monkey sperm mitochondria are tagged with ubiquitin after fertilization (Sutovsky et al., 1999), and in pre-implantation mouse embryos, paternal mitochondria are removed by a mitophagy pathway involving PINK1, Parkin and the alternative E3 ubiquitin ligase MUL1 (Rojansky et al., 2016). In *Drosophila*, ubiquitination of sperm mitochondria is not Parkin dependent, and the relevant E3 ubiquitin ligase remains to be discovered (Politi et al., 2014). Thus, although the process of sperm mitophagy seems widely conserved, the exact mechanism varies between species.

Another role of mitophagy has been identified in heart development. At birth, the fetal heart – up to then exposed to a hypoxic environment – switches its energy production from glycolysis to fatty acid oxidation. Intriguingly, Gong and colleagues (Gong et al., 2015) found that this perinatal switch in mice critically involves MFN2-Parkin-dependent mitophagy during the first 3 weeks after birth. The fetal mitochondria of mice expressing a dominant-negative form of MFN2, which lacks the PINK1 phosphorylation sites required for binding and mitochondrial translocation of Parkin, were unable to switch to fatty-acid-oxidation-dependent energy production. This resulted in cardiomyopathy-induced death at 7-8 weeks of age. These results strongly argue that mitochondria specialized for a certain mode of energy production are exchanged rather than transcriptionally reprogrammed and ‘remodeled’. Other examples of programmed mitophagy during cell differentiation processes have been observed during embryonic differentiation of retinal ganglion cells, and in macrophage activation and differentiation towards the M1 phenotype after lipopolysaccharides (LPS)/interferon γ stimulation (Esteban-Martínez et al., 2017). Although the perinatal heart switches its energy metabolism away from glycolysis and towards fatty acid oxidation, in the latter examples, the metabolic switch is towards glycolysis. Thus, mitophagy affects all kinds of metabolically ‘wired’ mitochondria.

During red blood cell differentiation, the mitophagy receptor NIX mediates mitochondrial removal (Sandoval et al., 2008; Schweers et al., 2007) in a manner independent of the conventional macroautophagy ATG5-dependent pathway. Rather, an alternative pathway that uses the Unc-51-like kinase 1 (ULK1) is involved (Honda et al., 2014). *Nix^−/−^* mice have shorter-lived erythrocytes that accumulate mitochondria, leading to anemia and erythroid hyperplasia (Diwan et al., 2007; Sandoval et al., 2008; Schweers et al., 2007), indicating that mitophagy is needed not only for development but also for cellular homeostasis in the adult.

### Mitophagy or UPR^mt^ as regulators of the innate immune response

Mitochondria are both targets of pathogen virulence factors and, at the same time, critically involved in mounting both innate and adaptive immune responses (Shpilka and Haynes, 2018). Many virulence factors and microbial toxins target host mitochondria for their abundance in essential nutrients. In addition, disrupting mitochondrial function also protects pathogens from mitochondrial ROS (Kwon et al., 2018; Tiku et al., 2020). The opportunistic bacterium *Pseudomonas aeruginosa*, for example, releases several toxins that dissipate the ΔΨ_m_ by directly interacting with the ETC or causing mitochondrial fragmentation (Kirienko et al., 2015; Manago et al., 2015).

Interestingly, a subset of bacteria – among them *P. aeruginosa* – induces the UPR^mt^ or mitophagy with favorable outcomes for the host (Kirienko et al., 2015; Liu et al., 2014; Pellegrino et al., 2014). Exposing worms to a purified bacterial siderophore induces mitophagy via iron chelation and thus promotes survival of the worms, revealing a role for mitophagy in the innate immune response (Kirienko et al., 2015). The explanation for this seemingly counterintuitive finding could be that mitophagy rids the cell of damaged ROS-producing mitochondria, which would otherwise lead to host cell death (pyroptosis) through the activation of the NLRP3 inflammasome (Bergsbaken et al., 2009; Lupfer et al., 2013; Zhou et al., 2011). Indeed, iron chelation did not only cause mitophagy but also significantly reduced ROS levels in the nematodes (Kirienko et al., 2015).

A UPR^mt^ initiated by mitochondrial stress in *C. elegans* also leads to the transcriptional induction of an innate immune response that partially overlaps with that seen upon *P. aeruginosa* infection (Pellegrino et al., 2014). Concordantly, *atfs-1* mutant worms are more susceptible to infection, while constitutive UPR^mt^ activation following expression of an *atfs-1* MTS mutant, which reduces mitochondrial import efficiency, prolonged survival due to accelerated clearing of *P. aeruginosa* (Pellegrino et al., 2014). These studies cumulatively suggest that the UPR^mt^ serves as a signaling hub enabling the detection of pathogens via ATFS-1; and the decision between UPR^mt^ and mitophagy either induces an antimicrobial transcriptional response that protects mitochondria or leads to the removal of the entire organelle to protect the host cell.

### ‘Piecemeal’ mitophagy

UPR^mt^, mitophagy and the factors initiating one over the other still need to be better defined. Moreover, mitophagy is energetically costly, explaining why mechanisms that preserve mitochondria or parts of them might have had an evolutionary advantage. Indeed, there is recent *in vitro* evidence for so-called ‘piecemeal’ mitophagy, in which specific parts of mitochondrial membranes or proteins are removed while others are left intact. Such a mechanism might oppose the detrimental translational effects of mtDNA mutations by selectively targeting faulty proteins for degradation. A screen for cargos within autophagosomes in HeLa cells, which combined enzyme-based proximity labeling with quantitative mass spectrometry, identified a novel LC3- and p62-dependent piecemeal mitophagy mechanism that is active under basal cell growth conditions and thus considered a housekeeping pathway (Le Guerroue et al., 2017). Localized induction of ROS generation through the expression of the mitochondria-targeted photosensitizer KillerRed, together with Parkin overexpression in HeLa cells, led to the recruitment of Parkin, ubiquitin and LC3 to the target site, followed by removal of the labeled mitochondrial ‘pieces’ (Yang and Yang, 2013).

Another mechanistically distinct piecemeal mitophagy pathway involves the formation of MDVs (Neuspiel et al., 2008). MDVs selectively transport oxidized mitochondrial components to the lysosome for degradation (Neuspiel et al., 2008; Soubannier et al., 2012; Todkar et al., 2021). PINK1, Parkin and VPS35 promote MDV formation and trafficking (Braschi et al., 2010; McLelland et al., 2014; Wang et al., 2016a). Interestingly, the protein product of *VPS35*, one of three genes associated with autosomal dominant PD (Zimprich et al., 2011), directly interacts with the mitochondrial fission protein DRP1 in the substantia nigra *in vivo*, and neurons expressing patient-specific *VPS35* variants showed a significant increase in mitochondrial fission. Subsequent experiments in dopaminergic neuroblastoma cells revealed that VPS35 regulates DRP1 complex turnover, which also involves its removal by MDVs (Wang et al., 2016a). Despite these intriguing studies, broader *in vivo* evidence of piecemeal mitophagy is still lacking.

Notably, the strong genetic interaction between *Vps35* and *Parkin* in *Drosophila* PD models is consistent with their cooperative action in MDV-mediated quality control (Malik et al., 2015). Surprisingly, the same study could not find an interaction between *Vps35* and *Pink1*, pointing to independent functions of Pink1 and Parkin. PINK1 also regulates the activity of complex I of the ETC through NDUFA10 phosphorylation, and the neurological defects in *pink^B9^*-null mutant *Drosophila*, a PD model, could be rescued by a phosphomimetic form of *NdufA10* (*ND-42*) (Morais et al., 2014; Pogson et al., 2014). As this function of Pink1 is independent of its role in mitophagy or Parkin, it suggests a two-hit hypothesis for PD involving independent functions of Pink1. Also, because both Parkin and PINK1 play a role in both MDV formation and mitophagy, it might also suggest that these pathways are not entirely separate.

## mtDNA as driver of the innate immune response

In a landmark study, Andrej Tarkowski's laboratory discovered that only mtDNA, but not nuclear DNA (nDNA), injected into the joints of mice induced arthritis via monocytes/macrophages, NF-κB and TNFα activation (Collins et al., 2004). Several disease states are suspected to result from the activation of the innate immune system in response to mtDNA released into the cytosol, such as systemic lupus erythematosus (SLE) and rheumatoid and inflammatory arthritis, cardiovascular and liver diseases, and age-related macular degeneration. Most of these associations are still correlative, and so we will focus here on general principles and a few examples. For a more extensive overview, we refer the reader to in-depth reviews (Riley and Tait, 2020; West and Shadel, 2017).

Over the course of evolution, mtDNA has transferred into the nuclear genome many times, with close to 900 nuclear mtDNA segments (NUMTs) (Lopez et al., 1994) described in humans, including nearly full-length insertions (Dayama et al., 2014; see also Box 3, ‘Technical challenges in studying mitochondria’). Still, mtDNA released into the cytosol due to mitochondrial stress, such as excessive ROS generation, elicits a type I interferon-mediated autoimmune response. Thus, specific characteristics of mtDNA residing in mitochondria must be the reason for it being sensed as ‘non-self’. These include the different methylation status of mtDNA and the fact that mtDNA often contains modified bases due to oxidative damage (Collins et al., 2004). In SLE, oxidized nucleoids (see Box 2) accumulate within mitochondria until the pro-inflammatory, interferogenic mtDNA is released (Caielli et al., 2016; Lood et al., 2016). While still debated, evidence points to mtDNA being minimally methylated (Ghosh et al., 2014; Hong et al., 2013; Mechta et al., 2017; Patil et al., 2019). Rather than at CpGs, both 5-methylcytosine (5mC) and 5-hydroxymethylcytosine (5hmC) occur randomly, predominantly outside of CpGs, reminiscent of plants and fungi (Bellizzi et al., 2013; Hong et al., 2013; Patil et al., 2019). Furthermore, mtDNA methylation seems to largely depend on cell type and differentiation stage, with human and mouse ESCs having the lowest levels (Bellizzi et al., 2013; Ghosh et al., 2016). Interestingly, although the cytosine methyltransferases DNMT1 and DNMT3A/B localize to mitochondria, non-CpG methylation persists in *Dnmt1^−/−^ Dnmt3a^−/−^ Dnmt3b^−/−^* triple-knockout mouse ESCs, indicating a role for other as yet unidentified DNA methyltransferases (Bellizzi et al., 2013). Moreover, triple-knockout ESCs maintain stem cell properties just like their wild-type counterparts (Tsumura et al., 2006). In contrast, acute knockdown of *DNMT3B* in the human mammary epithelial cell line MCF10A leads to profound reduction of mtDNA methylation (Patil et al., 2019). Another methylated DNA residue, *N*^6^-methyldeoxyadenosine (6mA) has recently been shown to be enriched in the mtDNA of human hepatoma cells to 1300-fold of the nDNA levels, with even higher levels under hypoxic conditions (Hao et al., 2020). 6mA might trace back to the bacterial origins of mtDNA, as it is widespread in prokaryotes, protecting their DNA from destruction. Hao and colleagues identified METTL4 as the methyltransferase responsible for ∼40% of mitochondrial 6mA (Hao et al., 2020). Interestingly, even though METTL4 can localize to mitochondria, it has neither an MTS nor a cleavable pre-sequence, indicating a potentially novel mechanism of translocation that awaits investigation.

Upon bacterial infection, bacterial DNA is recognized as a pathogen-associated molecular pattern (PAMP). Similarly, endogenous mtDNA that escapes into the cytosol is detected as a ‘foreign’ damage-associated molecular pattern (DAMP) by various pattern recognition receptors (PRRs) (Gong et al., 2020) (Fig. 5). Other DAMPs are mitochondrial double-stranded RNA (dsRNA), including RNA released in response to mtDNA double-strand breaks (Tigano et al., 2021), metabolites such as ATP, N-formyl peptides and even TFAM (see Box 2) (Rodriguez-Nuevo and Zorzano, 2019). Also, TFAM deficiency, via defective mtDNA packaging, causes mtDNA to escape into the cytosol (West et al., 2015).

The released mtDNA acts as a DAMP for cyclic GMP-AMP synthase (cGAS), a cytosolic PRR (Rongvaux et al., 2014; White et al., 2014). After recognizing double-stranded DNA (dsDNA) in the cytosol, cGAS synthesizes the second messenger 2′3′-cyclic GMP-AMP (cGAMP) that in turn binds to and activates stimulator of interferon response cGAMP interactor 1 (STING1) on the ER. STING1 initiates a phosphorylation cascade that activates transcription of a battery of interferon-stimulatory genes (ISGs) (Kumar, 2019). Although cGAS does not recognize RNA, it can still be activated by RNA viruses (Schoggins et al., 2014). For example, dengue virus induces the release of predominantly oxidized mtDNA, which in turn activates the cGAS-STING axis (Aguirre et al., 2017; Sun et al., 2017).

In the context of apoptosis, mtDNA enters the cytosol via Bak/Bax-driven OMM permeabilization and subsequent release of intermembrane proteins, including cytochrome c (Fuchs and Steller, 2015). Interestingly, the concomitant activation of the apoptotic initiator caspase-9 seems to actually dampen the cGAS-STING-mediated interferon response *in vivo* and *in vitro* (Rongvaux et al., 2014; White et al., 2014). Indeed, bone marrow chimeric mice generated by transplanting fetal liver cells from *Casp9^−/−^* mice or *Casp3^−/−^ Casp7^−/−^*mice, double mutant for two effector caspases activated by caspase 9, had elevated interferon-β serum levels (White et al., 2014), and type I interferon genes were upregulated in donor-derived mutant hematopoietic stem and progenitor cells (HSPCs). Functionally, the activated type I interferon response was accompanied by increased proliferation and functional exhaustion of those HSPCs in secondary transplants, and their multilineage reconstitution potential could be restored in mice doubly deficient for *Casp9* and type I interferon receptor (*Ifnar1*) (White et al., 2014). Explaining how these caspases might suppress type I interferon responses and thus why apoptosis is non-inflammatory, a recent study found that caspases 3 and 7 actually cleave and inactivate cGAS and proteins involved in other pattern recognition signaling pathways (Ning et al., 2019). In the context of viruses that evolved to express caspase inhibitors to circumvent host cell apoptosis, the cGAS-STING pathway enables the cell to mount a potent cell-intrinsic immune response (Rongvaux et al., 2014). Conversely, many viruses have evolved to target components of the cGAS-STING pathway for viral escape (Eaglesham and Kranzusch, 2020).

In addition to cGAS-STING activation, nucleic acids generated during mtDNA replication and transcription also engage other PRRs, including inflammasome components, Toll-like receptor 9 (TLR9) and retinoic acid-inducible gene-I (RIG-I; DDX58)-like receptors (RLRs) (Fig. 5). Different receptors recognize different nucleic acid species. For example, newly synthesized and oxidized mtDNA is sufficient to activate the NLRP3 inflammasome (Zhong et al., 2018), whereas the long dsRNA resulting from bidirectional transcription of mtDNA activates a type I interferon response via the RLR MDA5 (IFIH1) (Dhir et al., 2018). Patients with mutations in the ‘gatekeeper’ enzyme polynucleotide phosphorylase, which prevents the accumulation of mitochondrial dsRNA and its release into the cytosol, show upregulation of ISGs in peripheral blood and interferon in cerebrospinal fluid, as well as severe encephalomyopathy (Dhir et al., 2018; Matilainen et al., 2017; Vedrenne et al., 2012). Interestingly, mtDNA release can also trigger several PRRs simultaneously, such as in the case of dengue virus-induced mtDNA release, which can activate both cGAS and TLR9 in dendritic cells (Lai et al., 2018).

Owing to the involvement of the cGAS-STING pathway in eliciting a strong type I interferon response, efforts are ongoing to develop potent inhibitors against both human cGAS (Lama et al., 2019) and STING1 (Haag et al., 2018). cGAS-STING inhibitors could also help treat conditions that arise due to the proposed release of mtDNA. For example, genetically or pharmacologically blunting the cGAS-STING signaling pathway in mice ameliorates survival and recovery after myocardial infarction (MI) (King et al., 2017). However, while MI patients might have transiently increased plasma levels of mtDNA (Bliksoen et al., 2012; Wang et al., 2015), further studies will determine whether mtDNA causes the inflammatory response after MI. As a cautionary note, the quantitative polymerase chain reaction (qPCR) methods employed in those (and some other) studies do not control for nDNA contamination (see also Box 3), and, indeed, nDNA followed the same increase pattern (Wang et al., 2015), which is important as cytosolic nDNA can also activate the cGAS-STING pathway.

**Box 3. Technical challenges in studying mitochondria**

The study of mitochondria is hampered by several caveats. Some aspects to consider and examples of resulting controversies are the following:

- Integration of mtDNA into the nuclear genome as nuclear mtDNA segments (NUMTs) is a continuous evolutionary process (Mourier et al., 2001). Around 900 NUMTs have been identified in the human genome (Dayama et al., 2014), although this number varies depending on the method/parameters used. All parts of the mtDNA are represented in NUMTs, and some NUMTs are almost complete continuous mitochondrial genome insertions. Although, in rare cases, an NUMT insertion into a nuclear gene alters its expression and can cause disease (Turner et al., 2003), it is currently thought that NUMTs themselves are not expressed (Pozzi and Dowling, 2019). Polymerase chain reaction analysis using mtDNA-specific primers will also amplify NUMTs in case of nuclear contamination, which necessitates proper controls, for example, to conclude whether a specific mutation indeed resides within mtDNA.
- NUMTs also need to be considered in next-generation sequencing approaches (Wei et al., 2020), although the high copy number of mitochondria allows for sufficient coverage of the mitochondrial genome even with some nuclear contamination. In addition, detecting low-level heteroplasmy using next-generation sequencing methods can be confounded by sequencing errors (Bandelt and Salas, 2012; Li et al., 2016) or contamination with other samples (Just et al., 2014).
- Many heteroplasmy studies have involved cytoplasmic hybrid (cybrid) generation using Rho0 (ρ^0^) cell lines that are completely devoid of mtDNA (Wilkins et al., 2014). However, not only are the majority of ρ^0^ cell lines neoplastic, but the methods to deplete mtDNA are harsh, destroying OXPHOS so that, after selection, cells might have completely changed their metabolism. Conclusions using such systems thus need to be made with caution.
- Biochemically proving that proteins are localized to or excluded from mitochondria is complicated by the difficulty in obtaining highly purified mitochondrial fractions. During cell lysis, many proteins can artificially stick to the highly charged outer mitochondrial membrane (OMM) and therefore contaminate mitochondrial preparations. Furthermore, no universal criteria exist for the assignment of proteins to mitochondria (Krasich and Copeland, 2017). Irrespectively, a recent effort has manually annotated mitochondrial proteins (Rath et al., 2020).
- A defect in OXPHOS can be associated with both reduced and increased numbers of mtDNA molecules. On one hand, knockout of the mtDNA transcription factor *TFAM* reduces mtDNA copy number and impairs OXPHOS (Ekstrand et al., 2004; Larsson et al., 1998). On the other hand, amplification of mitochondria, which is assumed to compensate for defective OXPHOS, frequently occurs in affected tissues from patients with autosomal dominant optic atrophy (ADOA) or Charcot-Marie-Tooth type 2A (CMT2A) (Iommarini et al., 2012; Renaldo et al., 2012; Sitarz et al., 2012a; Sitarz et al., 2012b).
- MitoTracker dyes (e.g. MitoTracker Green) are fluorescent mitochondrial stains used to visualize and quantify mitochondria in live cells, due to their localization to mitochondria irrespective of ΔΨ_m_. Such dyes, for example, were critical in establishing that hematopoietic stem cells (HSCs) have lower mitochondrial mass (e.g. Simsek et al., 2010). However, the Snoeck group found that MitoTracker dyes can be poorly retained in mitochondria due to active xenobiotic efflux pumps in HSCs. A careful comparison of different methods to assess mitochondrial content led this group to conclude that HSCs, in fact, have a higher mitochondrial mass than their progeny, despite their lower mitochondrial respiration rate (de Almeida et al., 2017).

Mitochondrial stress and damage increase with age (see also Box 4, ‘A direct role for mitochondria in aging?’). The inflammatory response induced by mitochondria-derived molecules (‘mitoflammation’) has been implicated in chronic inflammation seen in age-related diseases, including PD and AD. Crossing Parkin homozygous mutant mice with ‘mutator mice’ (Box 4) elicits the PD phenotype with degeneration of dopaminergic neurons in the substantia nigra and motor coordination defects, which can be completely reverted by treating the mice with l-3,4-dihydroxyphenylalanine (L-DOPA) (Pickrell et al., 2015). Interestingly, mutator Parkin double-mutant mice also exhibited increased serum levels of mtDNA and of several cytokines, indicative of a type I interferon response. Similar phenotypes developed by subjecting Parkin mutant mice to exhaustive exercise and thus mitochondrial stress in the absence of *Polg* mutations. In both models, the immune response was cGAS-STING dependent. Increased cytokine levels and motor deficits were rescued by crossing the mice to *Sting1* mutants (Sliter et al., 2018). Interestingly, *Sting* did not contribute to the PD-like neuromuscular defects in *Pink1*/*parkin Drosophila* mutants (Lee et al., 2020a), even though the *parkin* mutant can mount an innate immune response (Greene et al., 2005). These results suggest that the fly immune response elicited by the *parkin* mutation might not be mediated by a cGAS-STING-dependent mechanism.

**Box 4. A direct role for mitochondria in aging?**

Mitochondrial dysfunction is one of the hallmarks of aging (Lopez-Otin et al., 2013). However, most data linking defective mitochondria with aging are correlative, and whether mitochondrial decline is a driving force of the aging process is still unclear.

- **ETC activity-dependent life span regulation** – On one hand, genetic and RNA interference (RNAi) screens in *C. elegans* uncovered that mutated or silenced components of the ETC or the ATP synthase markedly extended lifespan (Dillin et al., 2002; Feng et al., 2001; Lee et al., 2003). This was confirmed in *Drosophila* (Copeland et al., 2009). On the other hand, some ETC mutants are associated with reduced lifespan (Ishii et al., 1998; Kayser et al., 2004). One explanation for these discrepancies could be the degree of ETC inhibition: Rea et al. showed that, whereas lower doses of RNAi against essential ETC genes in *C. elegans* had mitohormetic lifespan-extending effects, high doses were lifespan shortening (Rea et al., 2007; Yun and Finkel, 2014). Along similar lines, genetically elevated levels of the ROS superoxide were necessary and sufficient for longevity in both worms and mice (Dell'agnello et al., 2007; Yang and Hekimi, 2010; Zarse et al., 2012). These mitohormetic effects underscore the notion of ROS as signaling molecules and contradict the older ‘free radical theory’ of aging that posits mitochondrially produced ROS to drive aging (Harman, 1956, 1992).
- **An impact of the UPR^mt^ on life span?** – In worms, RNAi-mediated knockdown of the nuclear ETC gene encoding cytochrome c oxidase subunit 5B (*cco-1*) in intestine or neuronal tissues promoted longevity (Durieux et al., 2011). Similarly, mild mitochondrial distress by ETC perturbation and concomitant ROS production in the muscle of flies could extend life span (Owusu-Ansah et al., 2013). Although both longevity responses required the mitochondrial unfolded protein response (UPR^mt^), in the fly model, the systemic repression of insulin signaling and mitophagy also contributed to the mitohormetic effect in the muscle.
  Intriguingly, reduction of *cco-1* in the worm neurons induced the UPR^mt^ in the intestine non-cell autonomously, and inhibiting the UPR^mt^ just in the intestine could not block life span extension, suggesting the existence of a ‘mitokine’ traveling from neuronal cells to the intestine. Subsequent studies showed roles for neuropeptides like FLP-2 and serotonin (Berendzen et al., 2016; Shao et al., 2016), and other mitokines have been identified since (Klaus and Ost, 2020).
  Whereas ETC perturbation in adult flies was sufficient to increase lifespan (Owusu-Ansah et al., 2013), in worms, lifespan extension required the inhibition of OXPHOS at specific developmental stages (Dillin et al., 2002; Durieux et al., 2011; Rea et al., 2007). Thus, different mitohormetic mechanisms seem to promote longevity in development and adulthood, the latter likely conferred by caloric restriction and insulin signaling (see discussion in Dillin et al., 2002). To explain the development-related effects on longevity, researchers proposed epigenetic mechanisms that perpetuate gene expression changes established during development into adulthood (Merkwirth et al., 2016; Tian et al., 2016). These depended on the JumonjiC (JmjC)-domain-containing histone demethylases acting on histone H3K27me2/3, a mark associated with inactive gene promoters. This mechanism might be conserved in mammals, as expression levels of the homologous demethylases *Phf8* and *Jmjd3* correlated positively with UPR^mt^-related genes and longevity in a large mouse panel (Merkwirth et al., 2016).
  Pharmacologically boosted NAD^+^ levels enhanced longevity in worms via the NAD-dependent deacetylase SIRT1/*sir-2.1* (Mouchiroud et al., 2013). At early adult stages, increased NAD^+^ levels induced the UPR^mt^, whereas slightly later, they also transcriptionally upregulated a ROS defense, suggesting a link between UPR^mt^ and the ROS defense pathway in mediating longevity. The prolonged longevity resulting from elevated NAD^+^ levels depended on *daf-16*, the *C. elegans* homolog of mammalian *FOXO3*, which encodes a critical transcription factor in the ROS defense and a deacetylation target of SIRT1 in mammals (Mouchiroud et al., 2013). Of note, Bennett et al. found that deletion of *atfs-1*, the central transcription factor of the UPR^mt^ in worms, failed to extend their lifespan (Bennett et al., 2014). More work will be needed to clearly define the precise modalities and the time- and tissue-dependent impact of the UPR^mt^ on longevity.
- **Mitochondrial DNA integrity and longevity** – Many different tissue types accumulate somatic mtDNA mutations with age and exhibit oxidized bases, base mismatches, strand breaks and deletions. After clonal expansion, these mutations lead to mosaic respiratory chain deficiency (Larsson, 2010).
  In humans, mtDNA point mutations accumulate with age in intestinal stem cells and their progeny in colonic crypts, coinciding with respiratory chain defects. However, a causal role in tissue aging remains to be shown (Taylor et al., 2003). ‘Mutator mice’ expressing a proofreading-deficient version of mitochondrial DNA Polγ (*PolgA^D257A^*) accumulate mtDNA mutations and develop premature aging phenotypes such as hair loss and graying, weight loss, kyphosis and osteoporosis, anemia and myeloid lineage skewing with lymphopenia (Ahlqvist et al., 2012, 2015; Kujoth et al., 2005; Norddahl et al., 2011; Trifunovic et al., 2004). Mechanistically, accumulating mtDNA point mutations in mutator mice destabilize ETC complexes I, III and IV (Edgar et al., 2009), but without increasing ROS production (Trifunovic et al., 2005). Nevertheless, treating mutator mice with antioxidants could rescue erythroid differentiation in embryos (Ahlqvist et al., 2012; Ahlqvist et al., 2015). In humans, *POLG* mutations are one of the most frequent causes of mitochondrial disease (https://tools.niehs.nih.gov/polg/), but patients do not present symptoms of premature aging. In addition, mice carrying a defect in mitochondrial genome maintenance exonuclease 1 (*Mgme1*), which in affected children causes mtDNA replication defects and a severe multisystemic mitochondrial disorder (Kornblum et al., 2013), accumulated mtDNA deletions similarly to mutator mice but did not develop symptoms of premature aging (Matic et al., 2018).

Apoptotic cell death aside, one unresolved question is how mtDNA escapes alive mitochondria. Several mechanisms have been implicated apart from apoptosis-mediated release. Recently, Kim and colleagues described that moderate, non-apoptotic stress triggers mtDNA release through pores formed by oligomers of the voltage-dependent anion channel (VDAC) proteins VDAC1 and possibly VDAC3. Patients with SLE, which is associated with a strong interferon signature (Ronnblom and Leonard, 2019), have upregulated VDAC1/3 expression. An inhibitor of VDAC oligomerization, VBIT-4, reduced lupus-like phenotypes and the levels of SLE-specific antibodies and mtDNA in the MpJ-*Fas^lpr^* mouse model, as well as the formation of neutrophil extracellular traps in low- and normal-density granulocytes in SLE patients (Kim et al., 2019). As this field of research is still in its infancy, many details remain to be learned regarding potential immunostimulatory roles of mtDNA during development, its cell-type specificity and, in particular, its causal role in disease and aging.

## mtDNA mutations and inheritance

### Heteroplasmy and the mitochondrial bottleneck

Although mtDNA encodes proteins essential for OXPHOS, it is not as protected from mutational events as nDNA. Rather, mtDNA has been found to evolve ten times more rapidly than nDNA (Brown et al., 1979; Kocher et al., 1989). These random mutations result in heteroplasmy, the presence of different mtDNA haplotypes in a cell (and individual), which can lead to mitochondrial diseases if the fraction of pathogenic mtDNA molecules surpasses a certain threshold (Fig. 6).

The continuous and random replication of mtDNA molecules, even in non-dividing cells, combined with a weak mtDNA damage repair system (see Box 2) are possible reasons for the high mutation rate of the mitochondrial genome. From an evolutionary point of view, a high mutational activity allows for diversity and adaptation to changing environments (Ruiz-Pesini et al., 2004) despite uniparental mtDNA inheritance and thus lack of recombination. At the same time, the inheritance of mtDNA mutations through the maternal germline without any reset mechanism could, in theory, lead to a mutational meltdown, also known as Muller's ratchet (Muller, 1964). Paradoxically, that has not been found to be the case: despite the high frequency of mtDNA mutations, few deleterious ones are transmitted to the offspring. A number of mechanisms contribute to this, such as a female germline mitochondrial genetic bottleneck, which results in random sampling and transmission of only a few mtDNA molecules. This sampling can also cause rapid shifts in heteroplasmy, which is how the mitochondrial genetic bottleneck was first proposed, after observing the rapid segregation of mtDNA D-loop sequence variants in several maternally related lineages of Holstein cows (Hauswirth and Laipis, 1982; Olivo et al., 1983). As the result of this bottleneck, a low-frequency allele can become completely fixed within a few generations. In humans, the mitochondrial genetic bottleneck was found to comprise about five mtDNA molecules per mitochondrion in primordial germ cells (PGCs) from healthy human female embryos between weeks 5 and 8 of gestation (Floros et al., 2018). Another study that extrapolated the size of the germline bottleneck from blood and cheek samples has arrived at similar results, with a range of seven to ten mtDNA molecules (Zaidi et al., 2019). Such tight bottlenecks can explain the very different heteroplasmy levels between sibling offspring from the same mother. Furthermore, mitochondrial bottlenecks both in the pre-natal (Cree et al., 2008; Floros et al., 2018; Freyer et al., 2012) and post-natal (Wai et al., 2008) germline have been described, at the level of the PGC and primordial follicle, respectively. Their sizes likely vary between individuals, as recently shown for a large cohort of 250 trio-families from all provinces in the Netherlands (Li et al., 2016). There are also species-specific size differences in mitochondrial bottlenecks; for example, mice exhibit considerably larger mitochondrial bottlenecks (∼200 mtDNA molecules) than humans (Jenuth et al., 1996). Following the germline bottleneck, oocyte specification coincides with the start of mtDNA replication, which rapidly increases the number of mtDNA molecules to at least 100,000 in the mature oocyte (Craven et al., 2010; Floros et al., 2018). This immense amplification requires mitochondrial biogenesis and functional respiration, which is achieved via an insulin-Myc feedforward loop initiated by transient activation of the c-Jun N-terminal kinase pathway (Wang et al., 2019).

Several not mutually exclusive mechanisms may explain how the mitochondrial genetic bottleneck is imposed. In one, stochastic segregation of a subset of mtDNA molecules into daughter cells passively reduces their number (Cree et al., 2008). However, it has been speculated that this finding could have been due to mtDNA counts from embryonic somatic cells, which have much lower mtDNA content, that were falsely identified as PGCs in flow cytometry analysis (Cao et al., 2009). An alternative mechanism could be the replication of only a subpopulation of mtDNAs (Wai et al., 2008) and their subsequent packaging into nucleoids to decrease the effective number of mtDNA segregation units (Cao et al., 2007) (see also Box 2). In the above-mentioned Dutch trio study, however, the best fitting bottleneck model involved individual mitochondria as a unit of segregation, rather than nucleoids.

Interestingly, analysis of a dataset of 30,506 mtDNA sequences representing the global population found that only 2.4% of nucleotides in human mtDNA show variation with an allele frequency of >1% (Wei et al., 2019). This observation implicates that random genetic drift, the primary force behind the mitochondrial bottleneck, could create PGCs with deleterious mutations that are then frequently selected against, resulting in an apparently much smaller variation. What leads to this negative selection against severe mitochondrial mutations? Addressing this issue experimentally has been difficult, as mammalian mitochondria cannot be simply transfected. ESC cybrids, which are mtDNA-depleted ESCs fused with enucleated donor cells carrying divergent or mutant mtDNAs, may provide a solution. These cybrids are injected into blastocysts to generate chimeric transmitochondrial mice, although this method is technically challenging and inefficient due to the low birth rates of mtDNA-mutant offspring (Fan et al., 2008; McKenzie et al., 2004). Of note, a simpler method for obtaining mice with heteroplasmic pathogenic mtDNA mutations has recently been developed. Breeding heterozygous mutator female mice, screening the founder individuals for mosaic cytochrome c oxidase deficiency in the colonic crypts, and sequencing the pathogenic mtDNA mutation can circumvent the problem that the strong purifying selection in the maternal germline poses for the study of mtDNA mutations (Kauppila et al., 2016).

Using the cybrid method, the Wallace laboratory introduced an mtDNA with two different mutations into mice: a heteroplasmic severe frameshift in the NADH dehydrogenase subunit 6 gene (*MT-ND6*) that, in a homoplasmic state, inactivates ETC complex I (Bai and Attardi, 1998), and a homoplasmic mild missense mutation in the cytochrome c oxidase subunit I gene (*MT-CO1*), which halves ETC complex IV activity (Acin-Perez et al., 2003; Kasahara et al., 2006). The severe *MT-ND6* mutation was actively selected against within four generations, while the weaker *MT-CO1* mutation persisted at the same level throughout multiple generations, even though the mice suffered from maternally inherited mitochondrial myopathy and cardiomyopathy (Fan et al., 2008). Similar results were obtained in experiments with mutator mice that were continuously backcrossed and analyzed for the transmission of random germline mtDNA mutations. mtDNA molecules with non-synonymous mutations in protein-coding genes, in particular in *MT-CO1* and *MT-CO2*, were strongly under-represented as early as in the second generation (Stewart et al., 2008). Thus, purifying (or negative) selection seems to eliminate highly deleterious mtDNA mutations. At the same time, however, it allows the transmission of milder mutations that are still pathogenic.

Important principles of selection have emerged from studies in *Drosophila*. In an elegant model, O'Farrell and colleagues (Xu et al., 2008) targeted the restriction enzyme *Xho*I to germline mitochondria for a single cut in a conserved region of *MT-CO1*, causing non-synonymous mutations. Using this strategy, 1% escaper progeny were recovered with *Xho*I-resistant point mutations, some of which with phenotypes recapitulating mitochondrial diseases, including neurodegeneration, muscle atrophy, growth defects, male sterility and a shortened life span. Age-related progressive diseases are often attributed to the accumulation of mtDNA mutational load and thus increased heteroplasmy in somatic tissues; however, in the *Drosophila* model, the mutant mtDNA was homoplasmic, indicating that the progressiveness and late onset of the phenotype is mutation dependent, rather than determined by the level of heteroplasmy. This method laid the groundwork for heteroplasmic flies carrying any combination of wild-type mtDNA, mtDNA harboring either mutant *MT-CO1* or *MT-ND2* alleles, or double-mutant mtDNA molecules (Ma et al., 2014). Whereas, for example, the pairing of the mitochondrial genome containing the *mt:CoI^T300I^* allele and wild-type genomes completely eliminated the mutant *mt:CoI^T300I^* genomes after 18 generations, two mitochondrial genomes with mutations in different genes that, by themselves, were selected against could complement each other and persist even after 50 generations. Thus, detrimental mutations can be stabilized through transmission due to intermolecular complementation. As the mechanism underlying the selection of mtDNA mutations, the authors speculated that in a heteroplasmic fly, mtDNA molecules supporting more robust OXPHOS could possibly be endowed with a small replicative advantage. Because the number of mitochondrial genomes increases exponentially during oogenesis, this small advantage would then be amplified, enabling their biased contribution to the oocytes' final mtDNA population (Ma et al., 2014). In a parallel study, Xu and colleagues indeed found that mtDNA replication is coupled to mitochondrial activity during *Drosophila* oogenesis (Hill et al., 2014). Hence, mtDNA that harbors the *mt:CoI^T300I^* mutation replicated less, and its elimination during purifying selection in the germarium, the tip of the fly's ovarium, coincided with the selective replication of wild-type mtDNA. Intriguingly, ectopic expression of the nuclear-encoded alternative oxidase that can bypass the cytochrome chain reactions could rescue viability of homoplasmic *mt:CoI^T300I^* mutant flies (Chen et al., 2015). In agreement with this, by conditionally expressing a dominant-negative form of ATP synthase and thus lowering ATP levels in wild-type mitochondria, another study was able to negatively select against wild-type mtDNA (Lieber et al., 2019) (see Box 5, ‘Strategies to shift heteroplasmy’).

**Box 5. Strategies to shift heteroplasmy**

- Mitochondrial heteroplasmy is the presence of more than one haplotype of mtDNA within a cell or individual. In both *C. elegans* and *Drosophila*, modulating various biological pathways reduces heteroplasmy. In a nematode model harboring a heteroplasmic 3.1-kb mtDNA deletion, Lin and colleagues reported a shift in heteroplasmy levels from 60% to 7% after impairment of the UPR^mt^ regulator *atfs-1*. In the same study, RNAi-mediated inhibition of mitochondrial fusion and fission, as well as that of *POLG* (*polg-1*) or *TFAM* (*hmg-5*), all reduced heteroplasmy (Lin et al., 2016). As another nematode model carrying heteroplasmic mtDNA mutations, ‘mutator worms’ carry a *POLG* mutation [*polg-1(srh1)*] analogous to that of mutator mice (see Box 4) and recapitulate major hallmarks of mitochondrial diseases. An RNAi screen identified the insulin growth factor-1/insulin signaling (IIS) pathway, mitophagy, autophagy, apoptosis and the UPR^mt^ as suppressors of their mobility defect. Interestingly, even though manipulation of the IIS pathway rescued the neuromuscular defect and increased the mtDNA copy number and the basal respiration rate in mutator worms, the mutation rate remained unchanged, suggesting the possibility of rescuing mtDNA diseases without correcting the true etiology (Haroon et al., 2018).
- Mitophagy has been further investigated as an obvious candidate mechanism to remove mitochondria carrying lethal mtDNA mutations. Interestingly, however, elimination of mutant mitochondria seems independent of canonical mitophagy involving Parkin (Ma et al., 2014) and Atg8 (Lieber et al., 2019; Zhang et al., 2019), but rather is driven by a specialized pathway previously found in red blood cell maturation and involving Atg1 and the *Drosophila* homolog of mammalian NIX (Lieber et al., 2019). In addition, PINK1 seems to play a mitophagy-independent role in marking mitochondria with mutant mtDNA by preferentially accumulating on them and inhibiting protein translation. Consequently, defective mitochondria are starved of nuclear-encoded mtDNA replication factors, which gives them a replicative disadvantage (Zhang et al., 2019). Replicative disadvantage of mutant mtDNA can also be achieved by deficiencies in the catalytic subunit of the nuclear-encoded mtDNA polymerase *POLG* itself. This link was uncovered in a *Drosophila* genome-wide screen for mutations that eliminated a detrimental mtDNA mutation that otherwise propagated for over 70 generations by selfish selection (Chiang et al., 2019). Accordingly, translation of fly PolG was also downregulated upon PINK1 accumulation on unfit mitochondria (Zhang et al., 2019).
- In somatic ovarian follicle cells, mtDNA mutations could be reduced by mitochondrial fragmentation (Lieber et al., 2019). Furthermore, in a model of mtDNA heteroplasmy in the *Drosophila* adult flight muscle, the deleterious mtDNA load could be decreased by several interventions that affect mitochondrial homeostasis, including knockdown of mitofusin and, interestingly, overexpression of *Pink1* or *parkin* (Kandul et al., 2016). Intriguingly, without intervention, autophagy eliminated mutant mtDNA from muscle tissue only to a limited extent, possibly explaining why pathogenic mtDNA accumulates in somatic tissues during aging (Kandul et al., 2016).
- Altogether, the discovery that numerous nuclear-encoded and pharmacologically targetable pathways control the propagation and transmission of deleterious mtDNA genomes bear potential for exciting prospective treatments of mitochondrial diseases.

Negative selection against mtDNA has also recently been directly visualized in the female *Drosophila* germline. Using heteroplasmic flies and an allele-specific fluorescence *in situ* hybridization (FISH) probe that hybridized to unique sequences in the D-loop region, the Lehmann and Hurd groups observed that negative selection of mutant *mt:CoI* mtDNA occurred during the differentiation of PGCs into cystoblasts in early oogenesis (Lieber et al., 2019). At this stage, which precedes the onset of mtDNA replication (Hill et al., 2014), mitochondrial networks fragment into smaller units (Chen et al., 2020; Cox and Spradling, 2003). Both the reduced number of mtDNA in fragmented mitochondria and the lack of mtDNA replication are thought to prevent mixing of mitochondrial genomes, as there is a lower chance that mutant and wild-type mtDNA are within the same mitochondrion. Fragmentation is indeed essential for negative selection, as mitofusin overexpression (Lieber et al., 2019) or knockdown of the fission factor *Fis1* (Chen et al., 2020) enrich for mutant mtDNA. Selection against deleterious mtDNA mutations in the *Drosophila* germline takes place at the organelle level, the mitochondrion, rather than at that of the cell, because no cell death could be observed in the germarium (Hill et al., 2014).

In contrast to negative selection of detrimental mitochondrial genomes, deleterious positive selection, known as ‘selfish selection’ has also been described. In experiments in flies, in which a mitochondrial genome with the lethal double mutant *mt:ND2^del1^*+*mt:CoI^T300I^* was paired up with a divergent *Drosophila* strain, the deleterious genome completely took over within approximately ten generations, even though the entire fly population died as soon as it became homoplasmic. This competitive advantage over the divergent mtDNA was mapped to the non-coding region that contains the origins of replication. This suggests that selfish positive selection can be initiated by sequence changes that create ‘super-replicators’, in contrast to purifying negative selection that minimizes changes in the coding regions of mtDNA (Ma and O'Farrell, 2016). In a disease context, these insights could have consequences for the selection of mitochondrial donors for mitochondrial replacement therapy (MRT), as even an mtDNA molecule conferring better OXPHOS can be outcompeted by a deleterious mtDNA that has acquired a selfish replicative advantage. A different type of selfish selection also occurs in somatic cells of *C. elegans* with mtDNA lacking four essential genes, which still can be maintained at ∼60-80% heteroplasmy due to a constitutively activated UPR^mt^. In *atfs*-1-depleted worms, the fraction of mutant mtDNA molecules decreased by about tenfold (Lin et al., 2016) due to Parkin-mediated mitophagy (Gitschlag et al., 2016). During human evolution, positive selection of mtDNA was likely a rare event: by analyzing non-synonymous mtDNA mutations over a 200,000-year time span across a reconstructed human phylogenetic tree, Cavadas and colleagues identified only one positively selected mtDNA mutation and instead found strong purifying selection, interrupted by two selection relaxations at the end of the last Ice Age and, before that, in the out-of-Africa human population expansion (Cavadas et al., 2015).

### Heteroplasmy and the threshold effect

Although initially thought to be a rare exception, deep-sequencing approaches detected low-level inherited and/or acquired heteroplasmy in many healthy human tissues (Ding et al., 2015; He et al., 2010; Li et al., 2015; Naue et al., 2015; Payne et al., 2013; Wei et al., 2019; Ye et al., 2014a,b), including oocytes (Floros et al., 2018). Although some of the results were argued to be technical false positives (Bandelt and Salas, 2012; Just et al., 2014), population-level mtDNA phylogenetic tree analyses suggest that widespread heteroplasmy might be genuine (Cavadas et al., 2015). Recently developed bioinformatics pipelines for single-cell genomic assays can detect somatic mtDNA mutations with heteroplasmy levels as low as 5%, and these mutations have been used as markers to trace clonally expanding populations during lineage differentiation processes (Ludwig et al., 2019). Such technologies have the potential to advance our understanding of heteroplasmy and its propagation in healthy individuals even further. In mice, the variance of mtDNA heteroplasmy linearly increases with maternal age in both oocytes and offspring (Burgstaller et al., 2018). Age-dependent increases in heteroplasmy have also been observed in human blood and cheek samples (Rebolledo-Jaramillo et al., 2014).

Hence, additional mechanisms are in place to prevent mtDNA mutations to phenotypically manifest themselves. In hybrid cells that are heteroplasmic for healthy and mutant mitochondrial genomes or myoblasts from patients suffering from myoclonus epilepsy and ragged-red fibers, the fraction of mutated mtDNA within cells must exceed a 65-90% threshold to manifest a respiratory chain deficiency (Boulet et al., 1992; Wallace, 1986). This means that, up to that threshold, wild-type mtDNA molecules can complement the defect in mutant mtDNA, so that most mtDNA mutations are functionally recessive (Filograna et al., 2019). mtDNA molecules are randomly replicated in a continuous fashion throughout the cell cycle (relaxed replication; see Box 2). In heteroplasmy, any mtDNA molecule could therefore by chance replicate more often than another, which could be the reason for *de novo* mtDNA mutations to clonally expand and cause a phenotype only later in life (Chinnery and Samuels, 1999). At the same time, mtDNA molecules – mutant or wild-type – are randomly distributed to daughter cells during cell division, further increasing the heterogeneity of heteroplasmy levels between different cells and tissues. Owing to the high copy number of mtDNA molecules per cell (see Box 2), a pathogenic mutation needs to go a long way until it reaches the threshold, again delaying fixation of one particular allele (Chinnery and Samuels, 1999). Of note, although humans live long enough to reach that threshold by random genetic drift, mathematical modeling showed that in short-lived animals such as mice or flies, random genetic drift is insufficient to account for the accumulation of mtDNA mutations (Kowald and Kirkwood, 2013), implying that selection mechanisms such as the selfish selection discussed above could be at work.

Intriguingly, not only the proportion, but also the absolute number, of healthy mtDNA molecules matters: infertility observed in mutator mice could be rescued merely by increasing the copy number of all mtDNA molecules, although the mutant mtDNA load and thus heteroplasmy levels remained unchanged (Jiang et al., 2017). In a subsequent study that used a different mouse model carrying a heteroplasmic mtDNA mutation in the tRNA^Ala^ gene, the same group found that increasing or decreasing mtDNA levels by modulating *TFAM* expression rescued or aggravated cardiomyopathy, respectively (Filograna et al., 2019). Furthermore, the effect of mtDNA copy number seems to depend on the proliferation rate of the tissue: in the fast-proliferating colonic epithelium, lowering the mtDNA copy number actually improved OXPHOS function over time because it speeds up clonal expansion of mutated mtDNA, ultimately selecting against cells with high levels of mutated mtDNA (Filograna et al., 2019).

### Uniparental inheritance

Paternal mtDNA is eliminated around the time of fertilization by several mechanisms. First, the ratio of mtDNA molecules contributed by human sperm and egg prior to fertilization is strongly skewed to ∼1 in 15,860 (Pyle et al., 2015). Second, negative selection mechanisms such as mitophagy eliminate any remaining paternal mitochondria so that paternal mtDNA is undetectable at the four- to eight-cell stage, at least in normally developing embryos (St John et al., 2000). Still, it is not entirely clear why uniparental maternal mtDNA inheritance was selected for during evolution. mtDNA is highly polymorphic, and coexistence of two potentially distant paternal and maternal mitochondrial genomes might result in unfit progeny. As genes encoding integral components of OXPHOS complexes have been retained in mtDNA, uniparental inheritance ensures that they can optimally co-evolve in response to changing environments (Wallace, 2007). Mice carrying two healthy mtDNA species that differ by 91 nucleotides were less active, had a slower metabolic rate, and learning, memory and behavioral deficits compared to their homoplasmic relatives. Consequently, these mice lost one of the mtDNA species over successive generations (Sharpley et al., 2012). Furthermore, as most OXPHOS complexes and the ATP synthase are encoded by both nDNA and mtDNA, uniparental inheritance could also have been favored due to optimal co-evolution of these two genomes to ensure the best performance without leakage of damaging ROS. Accumulating evidence from analyzing mtDNA haplotype and corresponding nuclear genetic ancestry in 12,162 individuals of European and Asian descent indeed points to co-evolution, in which newly identified mtDNA variants, in particular in OXPHOS genes, were selected for to match the nuclear ancestry (Wei et al., 2019). A similar nuclear-dependent selection of mtDNA variants has also been found in mice (Latorre-Pellicer et al., 2019; Latorre-Pellicer et al., 2016). Another hypothesis for maternal mtDNA inheritance posits that sperm mtDNA acquires too many deleterious mutations as by-products of the increased OXPHOS activity that sperm need to fertilize the egg (Ruiz-Pesini et al., 1998). Oocytes, in contrast, have been thought to be metabolically inactive, thus maintaining a low mtDNA mutational rate. However, more recent evidence indicates that oocytes might have a high OXPHOS activity (Dalton et al., 2014), raising doubt about this model.

The dogma of uniparental, maternal mitochondrial inheritance (Giles et al., 1980; Hutchison et al., 1974) has been challenged in different animal species, such as mice, *Drosophila* and partridge (for examples, see Gandolfi et al., 2017; Gyllensten et al., 1991; Wolff et al., 2013), and also in humans (Luo et al., 2018; Schwartz and Vissing, 2002; Wei et al., 2019), even though these occurrences seem rare (Pyle et al., 2015; Schwartz and Vissing, 2004). A suggested technical caveat in some of the studies could be the amplification of NUMTs during the deep-sequencing approach (Lutz-Bonengel and Parson, 2019; Wei et al., 2020). Determining the extent to which mtDNA inheritance might be affected by recombination between paternal and maternal mtDNA has a practical impact for genetic counseling and forensic studies. However, with the frequency of detected paternal mtDNA being this low, mtDNA molecules can be largely considered clonal between mother and offspring. In addition, the co-evolution of nuclear and mitochondrial genomes might need to be considered in the selection of mtDNA donors in mitochondrial replacement therapies to minimize nuclear-mitochondrial incompatibilities.

## Mitochondrial diseases

Mitochondrial diseases comprise a large variety of genetic human disorders caused by pathogenic mutations in both mtDNA and nDNA that are united by a primary defect in mitochondrial OXPHOS. Owing to the key role of mitochondria in energy metabolism, anabolic metabolic pathways and intracellular signaling, mitochondrial dysfunctions manifest as heterogeneous multisystem diseases, with a predominance in tissues particularly dependent on OXPHOS such as the brain, skeletal muscle, heart and eyes. However, any tissue can be affected, and disease onset can occur at any age, which often hampers diagnosis and has led to an underestimation of the prevalence (Table 2) (Gorman et al., 2016; Wallace, 2018). Mitochondrial diseases are the most common group of metabolic disorders, and they are also among the most common forms of inherited neurological disorders. mtDNA mutations could be the cause of ∼75% of adult-onset mitochondrial diseases based on a cohort study in the northeast of England (Gorman et al., 2015), but only of ∼20-25% of childhood-onset diseases (Lebon et al., 2003; Thorburn, 2004). The likely reason is that stronger mutations are likely to be removed by negative selection in the female germline, whereas relatively mild mutations escape that negative selection and manifest later in life. The majority of mitochondrial diseases in children is caused by autosomal recessive mutations in nDNA (Skladal et al., 2003).

**Table 2.**
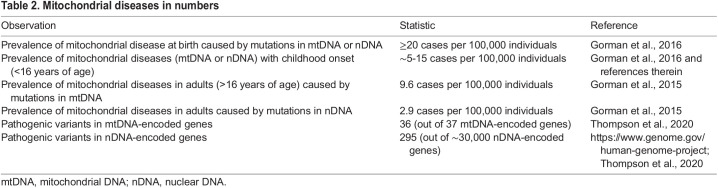
Mitochondrial diseases in numbers

The number of discovered mutations has increased at a fast pace since the identification of the first causal mutations in 1988, a point mutation in *MT-ND4* and large mtDNA deletions for Leber hereditary optic neuropathy (LHON) and mitochondrial myopathy, respectively (Holt et al., 1988; Wallace et al., 1988). While nDNA is inherited in Mendelian mode (autosomal or X-linked), mtDNA mutations are inherited by various mechanisms: the majority of mtDNA point mutations are maternally inherited, with 25% of them occurring *de novo*. Single large-scale mtDNA deletions (SLSMDs) almost always occur *de novo*. Owing to the mitochondrial bottleneck, recurrence rates between siblings are low for both types of mtDNA mutations, which is important for clinical counseling (Chinnery et al., 2004; Sallevelt et al., 2017). Heteroplasmic mutations often cause multi-system diseases with the level of pathogenic mtDNA heteroplasmy correlating with phenotype severity and varying in different tissues (Grady et al., 2018). Homoplasmic point mutations often cause a relatively mild defect that typically manifests only in one tissue. For example, mutations in LHON are frequently homoplasmic and although present in all tissues of the body only affect retinal ganglion cells, leading to blindness (Wallace et al., 1988). The reasons for this tissue selectivity are not known. Additionally, although all matrilineal members of a LHON pedigree carry LHON mutations, only some develop blindness. Factors like mtDNA background haplotype, mtDNA copy number, the nuclear background and environment could explain this incomplete penetrance (Bianco et al., 2017; Hudson et al., 2007; Kirkman et al., 2009).

The wide availability of next-generation sequencing technologies has spurred the discovery of mitochondrial disease caused by pathogenic variants in 116 novel nDNA-encoded genes between 2010 and 2017 alone (Frazier et al., 2019). Still, ∼60% of mitochondrial disease patients remain without a molecular diagnosis (Thompson et al., 2020). This is not surprising considering the 1123 nDNA-encoded proteins that reside in mitochondria (MitoCarta3.0 human inventory) (Rath et al., 2021), and many of them have only recently been associated with mitochondria. Proximity labeling proteomics approaches in living cells identified 62 new mitochondrial proteins, including those localized to the OMM (Hung et al., 2017, 2014; Rhee et al., 2013). Moreover, disease-causing variants in at least 15 genes that were previously not attributed a role in mitochondria have been identified (Stenton and Prokisch, 2018). Lastly, two recent clustered regularly interspaced short palindromic repeats (CRISPR)/Cas9 knockout and CRISPR interference (CRISPRi) screens found at least 39 genes with novel roles in mitochondria (Arroyo et al., 2016; Mendelsohn et al., 2018). Thus, more mitochondrial (disease) genes are likely to be discovered. In addition, with decreasing whole-genome sequencing costs, more non-coding variants are likely to be identified, although it will remain a challenge to assess their pathogenic potential.

Mitochondrial diseases were initially mostly attributed to genes encoding proteins directly involved in the biogenesis and function of the OXPHOS system, which alone consists of ∼100 proteins. However, unbiased sequencing technologies have increased the number of known genes that indirectly impair energy production (Frazier et al., 2019). These encode proteins with roles in mtDNA maintenance (Young and Copeland, 2016) or gene expression, mitochondrial dynamics, protein import/export, quality control and metabolism. Even though about two-thirds of known mutations affect genes encoding OXPHOS system components, mtDNA maintenance and expression (Frazier et al., 2019), the large number of affected processes also suggests that there are likely other vulnerabilities that can cause disease independent of just lowering ATP levels (Gorman et al., 2016) (see ‘Mitochondrial dynamics’ section).

Although mitochondrial diseases have been categorized as specific syndromes according to clinical features and age of onset (Table 3), many patients do not fit the classic presentation of a given syndrome, complicating diagnosis. Importantly, several different mutations in both nDNA and mtDNA can present as the same syndrome. Leigh syndrome, for example, is a collection of more than 75 monogenic diseases (Lake et al., 2016). Conversely, a single pathogenic lesion can be at the root of distinct clinical syndromes. For example, most children that survive Pearson marrow-pancreas syndrome are likely to develop Kearns-Sayre syndrome with the same causal SLSMD, however, accompanied by a decrease of pathogenic mtDNA molecules in blood and increase in muscle (McShane et al., 1991). Similarly, the m.3243A>G mutation in the mt-tRNA^Leu(UUR)^ gene *MT-TL1*, the most common disease-causing mtDNA mutation, with a carrier rate of 1 in 400 individuals, can manifest very differently with patients developing mitochondrial myopathy, encephalopathy, lactic acidosis and stroke-like episodes syndrome, maternally inherited deafness and diabetes, PEO or Leigh syndrome, among others (Nesbitt et al., 2013).

**Table 3.**
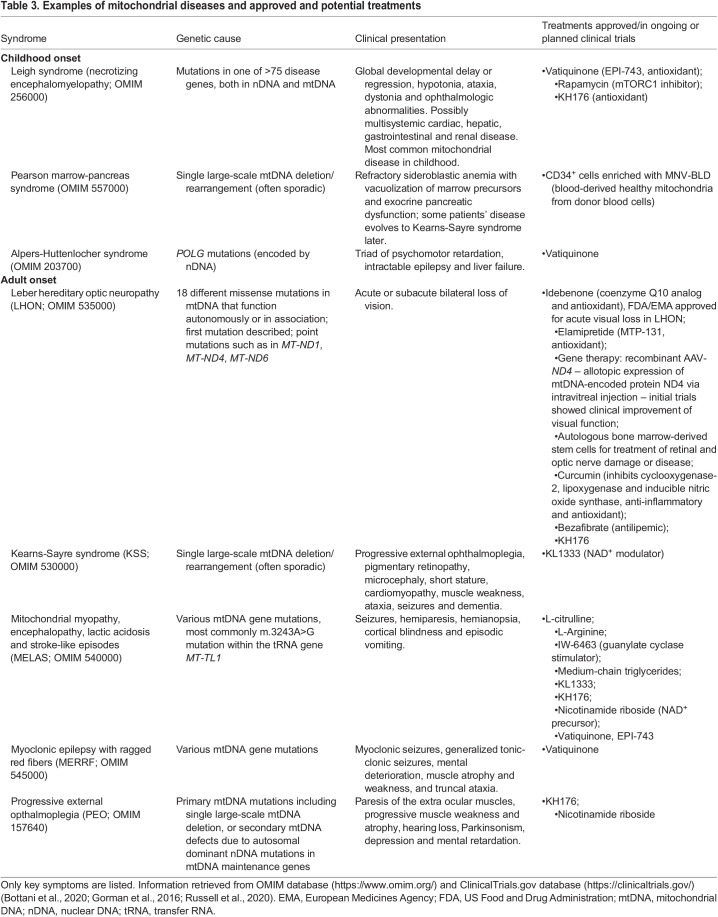
Examples of mitochondrial diseases and approved and potential treatments

Epigenetically induced changes in nuclear gene expression possibly contribute to the highly variable tissue phenotypes. This is interesting, as here, in contrast to the UPR^mt^, retrograde signaling is not initiated by mitochondrial stress but is rather due to heteroplasmy level. In patient-derived cybrid cell lines with varying heteroplasmy levels of the pathogenic m.3243A>G mutation, mutant mtDNA levels of 20-30% resulted in a low nuclear NAD^+^/NADH ratio, whereas 60-70% heteroplasmy led to elevated nuclear NAD^+^, paralleled by increased α-ketoglutarate (αKG) levels and decreased histone methylation, likely due to high activity of αKG-dependent histone demethylases. Finally, 90-100% mutant mtDNA decreased acetyl-CoA and histone acetylation levels, resulting in global transcriptional repression (Kopinski et al., 2019). Such changes may explain clinical phenotypes that drastically vary with the levels of heteroplasmy.

### Therapies for mitochondrial diseases

Morbidity and mortality from diseases caused by pathogenic mtDNA mutations remain high (Gorman et al., 2016; Russell et al., 2020). Current strategies to cure mitochondrial diseases aim at restoring mitochondrial function and include modification of the mtDNA, replacing mitochondria and adeno-associated virus (AAV)-mediated (e.g. AAV9) delivery of mtDNA-encoded genes. In addition, many small molecules have been developed to ameliorate mitochondrial function in a wide range of diseases (Table 3). For an excellent recent review of both pre-clinical studies and clinical trials using small molecules, see Bottani et al. (2020).

### Manipulating mtDNA

Approaches to specifically eliminate mutant mtDNA are based on the observation that a DNA double-strand break leads to the rapid degradation of the affected mtDNA molecule (Srivastava and Moraes, 2001; Tanaka et al., 2002). Through this process, specific depletion of mutant mtDNA could shift heteroplasmy. The first proof of concept that a mitochondrially targeted (mito) restriction endonuclease (RE) can effectively enter the mitochondrial matrix, cut and deplete the mtDNA harboring the restriction site, was made 20 years ago (Srivastava and Moraes, 2001). The pathogenic m.8993T>G mutation, which causes the progressive and severe neurodegenerative disorder Leigh syndrome (Table 3) creates an RE target site. Indeed, Tanaka and colleagues were able to shift the heteroplasmy caused by this pathogenic mutation towards the wild-type mtDNA in osteosarcoma cybrid cell lines by transfecting the mitoRE *Sma*I (Tanaka et al., 2002), the efficiency of which could be further improved by introducing the mitoRE *Xma*I (Alexeyev et al., 2008). *In vivo*, a heteroplasmy shift in striated muscle of neonatal mice harboring two polymorphic mtDNA sequence variants, NZB and BALB/c, was achieved by injecting the AAV-encoded mitoRE *Apa*LI that uniquely targets the BALB/c mtDNA variant (Bacman et al., 2012). Furthermore, injection of mRNA encoding the same mitoRE into mouse oocytes or zygotes resulted in offspring with marked reduction of the BALB/c mtDNA, pointing towards the feasibility of shifting mtDNA heteroplasmy to prevent mitochondrial disease inheritance (Reddy et al., 2015). Although these approaches show promise, they are limited by the requirement for an RE target site in the mutated mtDNA.

CRISPR/Cas9 has thus far not been successfully used to edit mtDNA due to the difficulties in delivering single functional guide RNAs into mitochondria (Hashimoto et al., 2015). As an alternative, zinc finger nucleases (ZFNs) and transcription activator-like effector (TALE) nucleases (TALENs) have been developed (Sun and Zhao, 2013). TALENs can be designed to target almost any DNA sequence, unlike ZFNs, which are artificial REs. Furthermore, some zinc finger domains lack specificity, resulting in more off-target cleavage (Sun and Zhao, 2013). By replacing the NLS with an MTS, Bacman and colleagues developed mitochondrial TALENs (mitoTALENs) to specifically target two different mutations in osteosarcoma cybrids, the SLSMD ‘common deletion’ m.8483-13459del4977, the most frequent mtDNA aberration, and the m.14459G>A point mutation (Bacman et al., 2013). Following this study, mitoTALENs and mitochondrially imported ZFNs were used to shift the heteroplasmy of different mutated mtDNAs in osteosarcoma cybrid cell lines, mouse oocytes and induced pluripotent stem cells (iPSCs) (Bacman et al., 2013; Gammage et al., 2016, 2014; Hashimoto et al., 2015; Reddy et al., 2015; Yahata et al., 2017; Yang et al., 2018). Finally, adenoviral delivery of mitoTALEN or mitoZFN was reported to shift heteroplasmy in a mouse model bearing the m.5024C>T mutation, which leads to tRNA^Ala^ instability that causes myopathies in humans (Bacman et al., 2018; Gammage et al., 2018).

In addition, two other enzymes were described to manipulate mtDNA. The MitoTev-TALE consists of a mitochondrially targeted sequence fused with a monomeric nuclease from T4 phage (I-TevI). Unlike TALENs and ZFNs that function as dimers, the MitoTev-TALE nuclease has the advantage of being effective as a monomer (Pereira et al., 2018). Recently, the Mougous and Liu groups described an interbacterial toxin named DddA that functions as a cytidine deaminase. The groups engineered two non-toxic split-DddA halves and fused each with an MTS and a TALE DNA-binding domain to target a specific sequence in the mtDNA. Binding of the two split-DddA halves to their proximal target DNA sequences and their colocalization converted CG to TA (Mok et al., 2020). Of note, this technology is limited to the editing of point mutations.

Despite the first evidence that mitochondrially targeted nucleases can be used to shift heteroplasmy emerging almost 20 years ago, this technology has so far not been translated to a clinical trial. In fact, besides the observed shift in heteroplasmy, different studies noted non-specific mtDNA depletion by mitoTALEN and mitoZFN. Indeed, transfection of mitoZFN in osteosarcoma cybrid cells first strongly depleted total mtDNA. Only a few days later, cells recovered their mtDNA with an associated heteroplasmy shift (Gammage et al., 2016). The same study also observed non-specific depletion of mtDNA by a mitoTALEN. A heteroplasmy shift after depletion of both wild-type and mutant mtDNA was also reported in iPSCs (Yahata et al., 2017), as well as in mice following an injection of an AAV-encoded mitoZFN (Gammage et al., 2018), hampering their translation to clinical use.

**Fig. 2.**
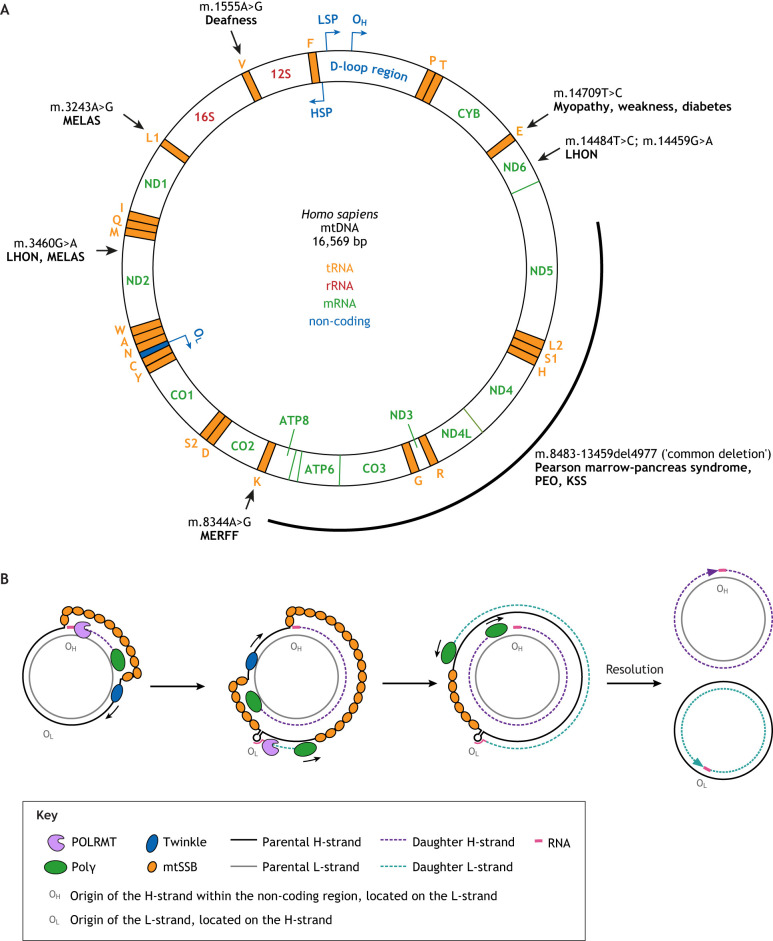
**Organization and replication of the human mitochondrial genome.** (A) Map of mtDNA. The black arrows point to the nucleotides affected in the indicated diseases; the black bar depicts the span of the ‘common deletion’ in mtDNA, which accounts for a third of Kearns-Sayre syndrome (KSS) cases (see Table 3). HSP, H-strand promoter; LHON, Leber hereditary optic neuropathy; LSP, L-strand promoter; MELAS, Mitochondrial myopathy, encephalopathy, lactic acidosis and stroke-like episodes; MERFF, myoclonic epilepsy with ragged red fibers; NCR, non-coding control region (also known as D-loop region); PEO, progressive external ophthalmoplegia. (B) Strand displacement model of mtDNA replication. After initiation of replication at O_H_ by an RNA primer transcribed by mitochondrial DNA-directed RNA polymerase (POLRMT), POLRMT is replaced by Polγ, which synthesizes the full-length nascent daughter heavy (H)-strand using the light (L)-strand DNA as the template, with the Twinkle helicase moving on the parental H-strand ahead of Polγ and mitochondrial single-stranded DNA (ssDNA)-binding protein (mtSSB) coating the displaced parental H-strand. Once Twinkle reveals O_L_, a stem-loop forms in the ssDNA of the parental H-strand, allowing the synthesis of a short RNA primer by POLRMT that is used to initiate synthesis of the daughter L-strand by Polγ using the displaced parental H-strand as a template. Twinkle is not required for L-strand synthesis because its template, the displaced H-strand, is unwound and coated with mtSSB. Primer removal and resolution of the hemicatenanes produces two double-stranded daughter mtDNA molecules.

In addition to their potential as a therapeutic strategy for mitochondrial diseases, mitoTALENs were employed to model the ‘common deletion’, a 4977 bp deletion linked to a number of mitochondrial diseases and cancer (Fig. 2A). By introducing various breaks in the mtDNA using mitoTALENs, Phillips and colleagues found that the common deletion was triggered by breaks near the 5′ repeats and was mediated by the mtDNA replication machinery (Phillips et al., 2017). Beyond editing mtDNA, a recent study demonstrated that mitochondrial transcripts can be specifically depleted. The recently discovered Cas13 family members are smaller than Cas9 and use only a single short CRISPR RNA to target RNA as their substrate (Abudayyeh et al., 2016; East-Seletsky et al., 2016; Konermann et al., 2018; Yan et al., 2018b). A *Drosophila*-optimized and MTS-tagged variant of the Cas13 family member Cas13d successfully targets transcripts of the OXPHOS genes *mt:CoI* or *mt:CoII*, the levels of which it could reduce by four- to fivefold (Huynh et al., 2020). In the future, mitochondrially targeted DNA and RNA nucleases and base editors should facilitate the study of mitochondrial diseases.

**Fig. 3.**
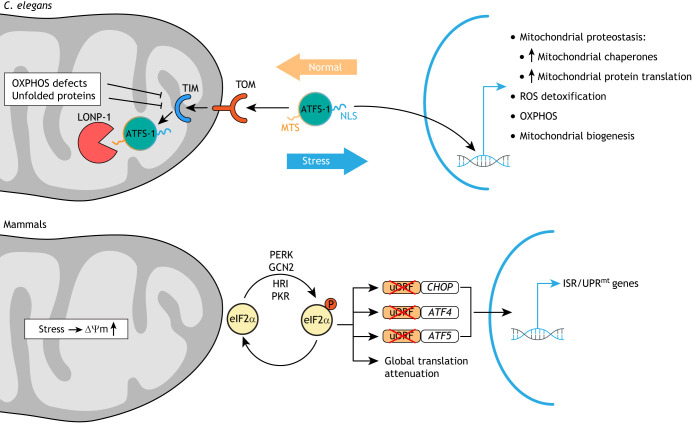
**Models of UPR^mt^ pathways in *C. elegans* and mammals.** In *C. elegans*, the mitochondrial unfolded protein response (UPR^mt^) is regulated by the subcellular localization of the transcription factor ATFS-1, which harbors both a mitochondrial targeting sequence (MTS) and a nuclear localization signal (NLS). ATFS-1 is normally efficiently imported into mitochondria through the TOM-TIM mitochondrial translocation complexes and degraded by the protease LONP-1. If ATFS-1 cannot be imported due to mitochondrial stress, ATFS-1 translocates, via the NLS, into the nucleus to activate a broad transcriptional stress response. In mammals, no direct homolog of ATFS-1 has been identified. Rather, the integrated stress response (ISR) is activated via the translation initiation factor eIF2α. Under mitochondrial stress conditions, eIF2α is phosphorylated by four different kinases (PERK, GCN2, HRI or PKR) that are activated by different stimuli. This leads to global translational attenuation, and, at the same time, translation of the transcription factors CHOP, ATF4 and ATF5. This occurs due to skipped translation of the upstream open reading frames (uORFs) that normally inhibit translation of the downstream *CHOP*, *ATF4* and *ATF5* coding sequences. OXPHOS, oxidative phosphorylation; ΔΨm, membrane potential.

**Fig. 4.**
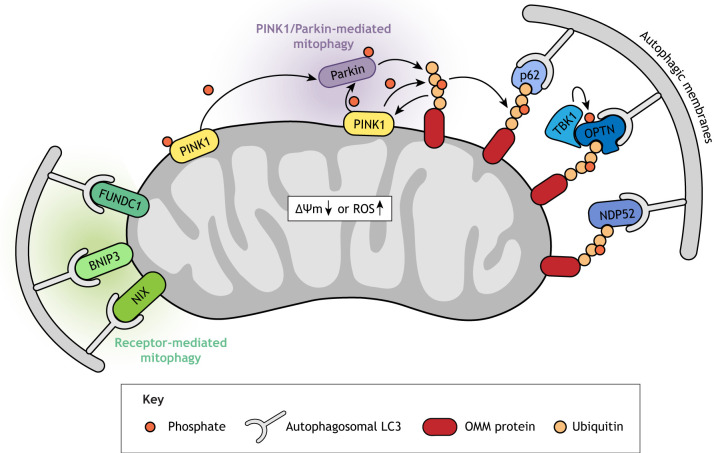
**Model of ubiquitin-dependent and -independent mitophagy pathways.** Mitochondrial stress stabilizes PINK1 on the outer mitochondrial membrane (OMM). PINK1 is activated by autophosphorylation and then phosphorylates Parkin and ubiquitin, both of which activate Parkin's E3 ligase activity. Parkin ubiquitinates several OMM proteins, and the resulting poly-ubiquitin chains in turn serve as additional phosphorylation targets for PINK1, creating a feed-forward loop. The phosphorylated poly-ubiquitin chains trigger the recruitment of the ubiquitin-binding adaptor proteins OPTN, NDP52 and p62, which initiate autophagosome formation by directly binding to the autophagosomal light chain 3 (LC3) protein through their LC-interacting region motifs. OPTN's affinity for ubiquitin chains is enhanced by its phosphorylation, and TANK binding kinase 1 (TBK1). Receptor-mediated mitophagy relies on various OMM proteins including BNIP3, NIX and FUNDC1, which directly interact with LC3 to mediate autophagosome formation.

**Fig. 5.**
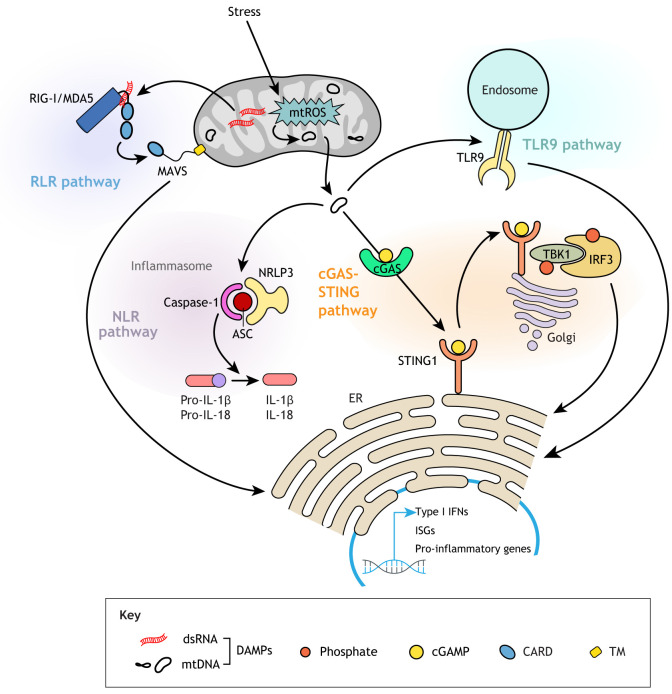
**Innate immune response pathways elicited by damage-associated molecular patterns (DAMPs).** Mitochondrial stress or Bak/Bax-driven OMM permeabilization can lead to release of mtDNA or dsRNA into the cytosol, triggering a cascade that results in activated expression of type I interferon and pro-inflammatory cytokine genes. Cytosolic mtDNA can bind the DNA-sensing protein cGAS that catalyzes the production of 2′3′-cyclic GMP-AMP (cGAMP), which in turn binds the adaptor molecule STING1 on the ER, activating TBK1. TBK1 phosphorylates and thus induces the translocation of the transcription factor IRF3 into the nucleus, where it activates type I interferon genes. mtDNA can also trigger a pro-inflammatory or type I interferon response via binding to Toll-like receptor 9 (TLR9) located on endosomes. In addition, mtDNA can be an endogenous agonist of cytosolic inflammasomes, multi-subunit complexes consisting of the receptor NLRP3 (or NLRC4, or AIM2), the adaptor ASC and the inflammatory cysteine protease caspase-1, which processes pro-IL-1β and pro-IL-18 into their mature forms. Double-stranded RNA (dsRNA) is recognized by the retinoic acid-inducible gene-I-like receptors (RLRs) RIG-I or melanoma differentiation-associated gene 5 (MDA5), which bind to mitochondrial antiviral signaling protein (MAVS) through homotypic caspase activation and recruitment domain (CARD)-CARD interactions. MAVS then recruits various molecules to transduce a downstream signal to the nucleus, resulting in the activation of target genes. ER, endoplasmic reticulum; IFN, interferon; ISG, interferon-stimulatory gene; mtROS, mitochondrial reactive oxygen species.

**Fig. 6.**
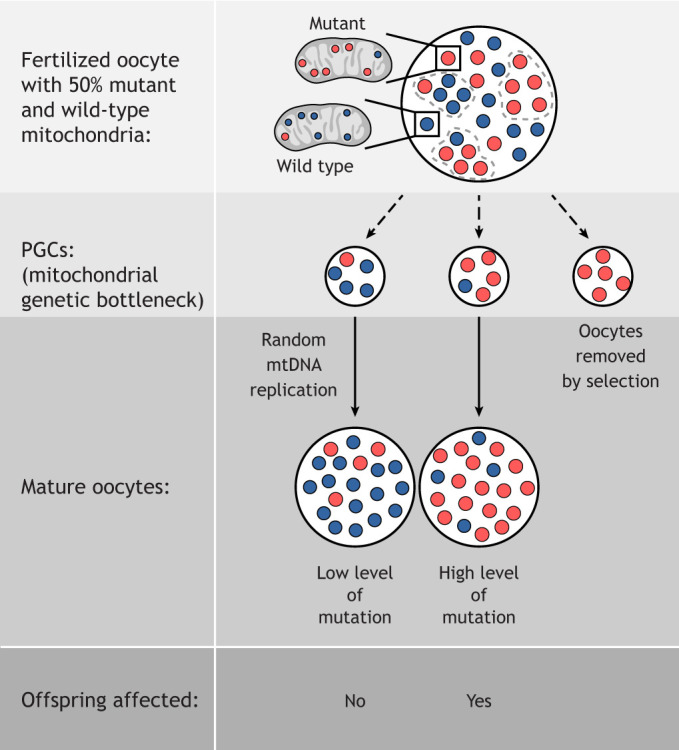
**The mitochondrial bottleneck in the female germline and its consequences for the offspring.** The penetrance of phenotypes depends on the level of heteroplasmy, which represents mutant mtDNA load. A threshold of 65-90% mutant mtDNA needs to be surpassed for a respiratory chain deficiency to manifest. PGC, primordial germ cell.

### Artificial mitochondrial transfer

Artificial mitochondrial transfer (AMT) enriches recipient cells with healthy mitochondria from donor cells. Physiological mitochondrial transfer, also referred to as horizontal transfer, has been demonstrated to occur *in vitro* and *in vivo* (Islam et al., 2012; Rustom et al., 2004; Spees et al., 2006). In contrast, AMT was achieved much earlier, when in 1982 Clark and Shay produced antibiotic-resistant mammalian cells by purifying and transferring resistant mitochondria into sensitive recipient cells (Clark and Shay, 1982). More recent pre-clinical studies show promise for AMT in myocardial ischemia-reperfusion injury (McCully et al., 2009) and neuronal degeneration (Kuo et al., 2017), including PD (Chang et al., 2016). Myocardial ischemia leads to significant damage in mitochondrial structure and function, which persists into reperfusion and severely compromises cell viability and thus post-ischemic recovery. Myocardial ischemia-reperfusion injury resulting from cardiac surgery is associated with poor clinical outcomes (Wang et al., 2018b). After promising pre-clinical studies in rabbit and pig models (Kaza et al., 2017; Masuzawa et al., 2013), McCully and colleagues treated the first five pediatric patients with myocardial ischemia-reperfusion injury after cardiac surgery for congenital heart disease with an AMT-based therapy using healthy autologous mitochondria from non-ischemic skeletal muscle, without short-term complications due to injection of mitochondria (Emani et al., 2017). In a retrospective study, ten patients showed improved cardiac outcomes (Guariento et al., 2020) after AMT, warranting expansion into a clinical trial that is still ongoing. Another clinical trial is treating patients with Pearson marrow-pancreas syndrome with an AMT therapy that deploys autologous hematopoietic stem cells enriched with healthy mitochondria derived from maternal donor blood cells (Table 3) (Jacoby et al., 2018).

### MRT

MRT is a reproductive medicine approach that was initially developed for women diagnosed with mitochondrial disease, but has been also used to treat infertility. Two main methods of MRT have been described. In the pronuclear transfer method, pronuclei from the fertilized egg of an affected woman are transferred to an enucleated egg from a healthy donor. The second method, known as maternal spindle transfer (MST), is based on the observation that oocytes arrest in metaphase of meiosis II until their fertilization, which allows the transplantation of the meiotic spindle with the chromosomes attached to it in this time window. While MST has successfully enabled a female carrier of Leigh syndrome to give birth to a healthy boy (Zhang et al., 2017), MRT, or commonly called the ‘three-parent’ technique, remains unauthorized in many countries, including the USA (https://www.fda.gov/vaccines-blood-biologics/cellular-gene-therapy-products/advisory-legal-restrictions-use-mitochondrial-replacement-techniques-introduce-donor-mitochondria). Apart from ethical concerns, a variable amount of mutant mtDNA can be carried over in both pronuclear and MST methods. For example, tissues tested in the above-described MST-derived neonate still had an mtDNA mutation load of 2.36-9.23%. In some cases, even a very low number of mtDNA molecules transferred from the egg of the affected mother, possibly due to specific polymorphisms in their D-loop region and/or sequence-independent mechanisms, could have a replicative advantage over the donor mtDNA, leading to a gradual loss of the donor mtDNA and reversal to the maternal genotype (Hudson et al., 2019; Kang et al., 2019; Kang et al., 2016).

### Gene therapy

Single point mutations in the mitochondrial gene encoding ND4 (MT-ND4), a subunit of complex I, result in OXPHOS defects and are associated with LHON (Table 3). Patients with LHON have normal life expectancy but lose their vision during their second or third decades of life. In 2002, Guy and colleagues recoded *ND4* as a nuclear gene and added an MTS to allow mitochondrial import of the protein. This allotopic expression of ND4 rescued deficient OXPHOS of heteroplasmic cybrid cells carrying the m.G11778A mutation, the most common LHON-causing mutation worldwide (Guy et al., 2002). After assessing the safety and efficacy of intravitreal injection of recombinant AAV-*ND4* in rodents and non-human primates (Cwerman-Thibault et al., 2015; Guy et al., 2009; Koilkonda et al., 2014), this gene therapy is currently pursued in human clinical trials by three investigator groups (Guy et al., 2017; Wan et al., 2016; Yu-Wai-Man et al., 2020; Yuan et al., 2020). One of these, a global, multi-center trial, reported promising phase III results (Yu-Wai-Man et al., 2020). Of note, all studies observed an improvement of visual function in the untreated eye after unilateral intravitreal administration of the viral vector. As a possible mechanism, transfer of the viral vector to the optic nerve of the contralateral non-injected eye was demonstrated in non-human primates (Yu-Wai-Man et al., 2020).

Intriguingly, expression of the *Saccharomyces cerevisiae* NADH dehydrogenase type 2 (Ndi1), the single-protein substitute for complex I that catalyzes the electron transfer from NADH to ubiquinone without pumping protons across the IMM, completely rescued the ΔΨ_m_ in a *nuo-1* missense mutant *C. elegans* model of ETC complex I deficiency. This mitigated many of the associated phenotypes in the worms, including shortened survival in the presence of oxidative stress and premature aging (DeCorby et al., 2007). Dysfunction of complexes I and III, accompanied by impaired OXPHOS and mitochondrial oxidative stress, mediates demyelination of optic nerve axons and retinal ganglion cells (RGCs) in the experimental autoimmune encephalomyelitis (EAE) mouse model of multiple sclerosis (MS). Notably, intravitreal injection of an AAV expressing yeast Ndi1 reduced oxidative stress, ameliorated the loss of optic nerve axons and RGCs, and improved visual function (Talla et al., 2020, 2013). In the same model, complex I and III function could be completely rescued by intravitreal injection of an AAV overexpressing mitochondrial HSP70 (mtHSP70), which also prevented RGC loss and suppressed loss of vision (Talla et al., 2014). These studies open up a new avenue in the treatment of optical neuritis associated with MS.

Finally, gene therapy was also successfully employed to rescue the cardiac failure observed in the Friedreich ataxia (FRDA) mouse model. FRDA is caused by a reduced expression of the nuclear-encoded mitochondrial protein frataxin (FXN). Expression of human FXN upon intravenous injection of an AAV-*FXN* vector in the mouse model extended lifespan, improved cardiac function and prevented peripheral sensory neuropathy (Gerard et al., 2014; Perdomini et al., 2014; Piguet et al., 2018). Further experiments will determine whether this new gene therapy can be translated into patients.

## Outlook

Research on mitochondria has recently exploded: of the 369,798 papers published since 1913, two-thirds were published within the past 20 years (PubMed search on 23 May 2021 for ‘mitochondria’ or ‘mitochondrial’). Investigation of processes such as the UPR^mt^, mitophagy and the role of mtDNA in the innate immune response have opened up entirely new research areas. Next-generation sequencing technologies have not only allowed us to revisit questions about our ancestry but also to identify more causal mutations that drive mitochondrial diseases. A common theme is that mtDNA, mitochondrial dynamics and quality control themselves can be responsible for alterations in mitochondrial metabolism and disease phenotypes. Still, we are only at the beginning of grasping the complexity of this organelle. We need to better understand how mitochondria integrate as ‘signaling hubs’ with the rest of the cell, that is, how the once symbiotic relationship has evolved. The crosstalk between mitochondria and the nucleus, not just as a cellular response to acute mitochondrial stress but also an adaptation of the nuclear epigenetic landscape to changes in metabolism due to mtDNA variants, deserves revisiting. In this regard, we might need to be more careful in interpreting data that are generated with mouse models harboring mtDNA variants (Fetterman and Ballinger, 2019; Wei et al., 2019). Also, even though the mtDNA molecules of most higher organisms have been sequenced, the list of new mtDNA-encoded peptides is still growing (Kim et al., 2017).

Elucidating mitochondrial biology in native physiological or pathological contexts rather than in cell culture or *in vivo* systems involving overexpression or artificial reporters remains a challenge. New technologies that directly target mtDNA will soon allow researchers to replace mouse models that have been generated from cybrid cell lines with models harboring variants in their endogenous mtDNA. Furthermore, considering how functionally interconnected mitochondria are with the rest of the cell and, for that matter, the entire organism, it remains a formidable task to distinguish direct from indirect effects. In this context, we only poorly understand how mitochondria communicate among themselves within a cell, between cells and even between tissues. How many mitokines exist and do they exert specificity (Klaus and Ost, 2020)? Is intermitochondrial communication within cells akin to microbial quorum sensing (Williams and George, 2019)? Although mitochondrial fusion is readily observed in cultured cells, it occurs less frequently in differentiated tissues such as striated muscle, where mitochondria are spatially more confined (Eisner et al., 2017, 2014; Huang et al., 2013), and mitochondrial communication might more frequently involve nanotunnels, membranous tubular protrusions emanating from mitochondrial surfaces (Vincent et al., 2017). Furthermore, interorganelle communication between mitochondria and organelles other than the ER are not very well understood. Mitochondria play critical roles in cell fate decisions, stemness and differentiation, and mitochondrial dysfunction might underlie the most common neurodegenerative disorders and diseases of aging. Thus, understanding mitochondrial biology has enormous potential to uncover new therapeutic strategies for many life-threatening diseases and increase overall quality of life.
